# Easily accessible 2^A^-*O*-Ts-β-cyclodextrin *via* efficient aqueous conditions

**DOI:** 10.1039/d6ra05416a

**Published:** 2026-08-17

**Authors:** Golfo G. Kordopati, Maria Tsouna, Gerasimos M. Tsivgoulis

**Affiliations:** a Department of Chemistry, University of Patras 26504 Greece gkordopati@upatras.gr g.tsivgoulis@upatras.gr

## Abstract

A simple and efficient method for the synthesis of 2^A^-*O*-Ts-β-cyclodextrin (2^A^-*O*-Ts-β-CD), an important intermediate in cyclodextrin chemistry, is reported. The method employs 1-tosyl-1*H*-1,2,4-triazole (Ts-1*H*-Trz) as a mild tosylating reagent under aqueous conditions, using NaHCO_3_ as a weak base. The methodology avoids strictly anhydrous conditions, eliminates the use of hazardous reagents such as NaH, and allows commercially available hydrated β-cyclodextrin to be used directly without prior dehydration. Reaction parameters, including co-solvent composition, temperature, base, and the nature and stoichiometry of the tosylating reagent, were evaluated to identify efficient conditions. A 1 : 1.6 (v/v) water/1,4-dioxane mixture at 50 °C provided the best balance between β-cyclodextrin solubility and suppression of side reactions. Among the tosylating reagents examined, namely tosyl chloride (Ts-Cl), 1-tosyl-1*H*-imidazole (Ts-Im), and Ts-1*H*-Trz, the latter exhibited the highest efficiency, affording 2^A^-*O*-Ts-β-CD in 43% isolated yield with a decrease in the relative ESI-MS signal of the corresponding by-product ions. NMR investigations were carried out to probe the effect of host–guest interactions between β-cyclodextrin and Ts-1*H*-Trz on the reaction outcome. The developed protocol provides a safer, more sustainable, and operationally straightforward approach for the preparation of 2-monotosylated β-cyclodextrin. The isolated yield obtained in aqueous medium exceeds those reported for previous aqueous methods and is comparable to that achieved by the most efficient conventional non-aqueous procedures.

## Abstract 2

Efficient preparation of 2^A^-*O*-Ts-β-cyclodextrin in water under semi-optimized conditions. NMR studies do not provide evidence for a stable β-cyclodextrin-tosylating reagent inclusion complex under the conditions examined.

## Graphical abstract

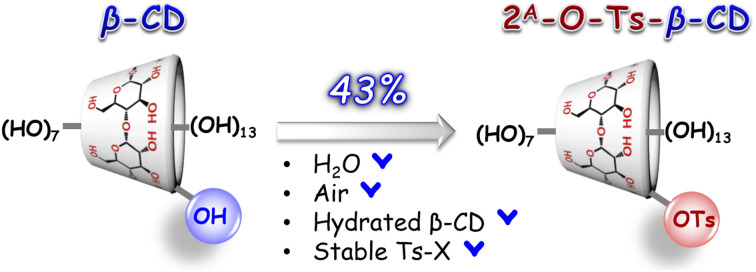

## Introduction

Cyclodextrins (CDs) are cyclic oligosaccharides composed of α-d-glucopyranose units linked by α-1,4-glycosidic bonds, and they represent one of the most important classes of molecules in supramolecular chemistry. Cyclodextrins can form inclusion complexes with a wide variety of substrates^1–4^ of different structures, polarities, and lipophilicities, while maintaining biocompatibility.^5,6^ Hence, they have been repeatedly used to overcome the solubility limitations of many poorly soluble organic molecules.^7,8^

Derivatization of CDs can yield molecules with enhanced or modulated properties relative to their unmodified counterparts. However, achieving precise cyclodextrin substitution remains a challenging task. Even in the case of the smallest α-cyclodextrin, there are 18 hydroxyl groups, distributed among three distinct types C2–OH, C3–OH, and C6–OH based on their positions on the glucopyranose ring. In β- and γ-cyclodextrins, the total number of hydroxyl groups increases further to 21 and 24, respectively. The C6–OH groups are primary hydroxyls located on the primary face of the cyclodextrin molecule. Owing to their lower steric hindrance and higher nucleophilicity, they are generally more reactive toward electrophilic reagents.^9^ In contrast, the C2–OH and C3–OH hydroxyls are located on the secondary face.^10^ Among these hydroxyl groups, the C2–OH groups exhibit higher acidity (p*K*_a_ ≈ 12.2)^11^ due to the electron-withdrawing effect of the adjacent glycosidic oxygen atoms, facilitating their deprotonation under basic conditions and contributing to their preferential functionalization under suitable reaction conditions.^12,13^ Selective mono-functionalization at specific hydroxyl positions is therefore commonly achieved by exploiting differences in hydroxyl group acidity, nucleophilicity, and steric accessibility, together with careful control of reaction parameters such as the choice of reagent, base, solvent, stoichiometry, and temperature.^9^ These factors enable preferential modification at a specific hydroxyl position while minimizing undesired multiple substitutions. Selective chemical modification of the secondary hydroxyl groups is more difficult than that of the primary ones due to their lower reactivity and greater steric hindrance.^10^

Among cyclodextrin (CD) derivatives, tosylates are valuable synthetic intermediates due to the excellent leaving-group ability of the tosylate group, which facilitates nucleophilic substitution by a broad range of nucleophiles. Accordingly, tosylated CDs are widely employed as versatile precursors in the synthesis of CD-based molecules designed for either biological^7,14–21^ or technological^18–24^ applications. In particular, 2^A^-*O*-Ts-β-cyclodextrin (2^A^-*O*-Ts-β-CD) represents a key intermediate in cyclodextrin chemistry, as the tosyl group at the C-2 position provides a versatile reactive site that enables nucleophilic substitution either directly or through the formation of a reactive 2,3-*mannoepoxy*-β-cyclodextrin intermediate,^25^ thereby allowing access to a wide range of regioselectively functionalized β-cyclodextrin derivatives, including azido, amino, thio, and other substituted analogues.^26,27^ Such derivatives have been extensively employed in the development of cyclodextrin-based conjugates and functional materials for applications in click chemistry, drug delivery, molecular recognition, and supramolecular assemblies.^28–30^ Several studies in the literature describe the synthesis of either 6^A^-*O*-Ts-β-cyclodextrin (6^A^-*O*-Ts-β-CD) or 2^A^-*O*-Ts-β-cyclodextrin (2^A^-*O*-Ts-β-CD) under very diverse reaction conditions, generally affording only moderate yields (typically <42%), highlighting the synthetic challenges associated with their preparation. Factors such as selectivity, reproducibility, solubility, and product purity are among those that render cyclodextrin chemistry particularly demanding.

In particular, for the synthesis of 2^A^-*O*-Ts-β-CD, Ueno and Breslow^31^ first reported the successful sulfonylation and isolation of the product in 10% yield in 1982, using 3-nitrophenyl 4-toluenesulfonate as the sulfonating reagent in carbonate buffer/DMF. By replacing the carbonate buffer with NaH in dry DMF (anhydrous conditions) and using tosyl chloride (Ts-Cl) as the tosylating reagent, Rong and D'Souza^32^ reported a 30% yield (isolated as a mixture), while Law *et al.*^33^ obtained a 42% yield when 1-tosyl-1*H*-1,2,4-triazole (Ts-1*H*-Trz) was used as the tosylating reagent. In a different approach, a 32% yield was obtained when Ts-Cl in DMF was added to a CD cyclic tin intermediate.^34^ Using 1-tosyl-1*H*-imidazole (Ts-Im) in DMF in the presence of molecular sieves and without any added base, a 36% yield was achieved; however, this reaction proceeded very slowly, requiring several days (∼50 h) for substantial conversion.^35^ In 2007 K. Martina *et al.*^36^ achieved monotosylation using the same conditions but with ultrasound irradiation, obtaining 2^A^-*O*-Ts-β-CD at a 40% yield within a very short reaction time (1 h). Using the same tosylating agent in DMF as solvent and Cs_2_CO_3_ as catalyst, Yu *et al.* selectively sulfonylated the C2–OH group, with a 32% yield.^37^ Wang *et al.* reported a 28% yield^38^ using a combination of Ts-Im as the tosylating agent and carbonate buffer in DMF. In 2007, M. Strerath *et al.* reported a 23% yield of 2^A^-*O*-Ts-β-CD under aqueous conditions using TsCl as the tosylating agent in a 1 : 1 (v/v) 1,4-dioxane/H_2_O mixture and Na_2_CO_3_ as the base.^39^ The yield was later increased to 34% in 2011 when Ts-Cl was replaced by Ts-Im.^40^ Additionally, the use of Ts-Im and KOH as the base afforded 2^A^-*O*-Ts-β-CD in a significantly improved yield (53%), although mechanochemical synthesis using a ball mill was required.^41^

Recently, our group reported the synthesis of 2^A^-*O*-Ts-β-CD in 40% yield using NaH in anhydrous DMF and dried β-CD.^42^ The factors affecting the reaction were systematically investigated and optimized, thereby addressing reproducibility issues commonly encountered in cyclodextrin functionalization reactions. However, this method requires strictly anhydrous conditions and the use of the highly reactive reagent NaH. Consequently, the reaction must be performed under an inert, moisture-free atmosphere using dried solvents, and the commercially available hydrated form of β-CD cannot be used without prior drying.

**Scheme 1:**
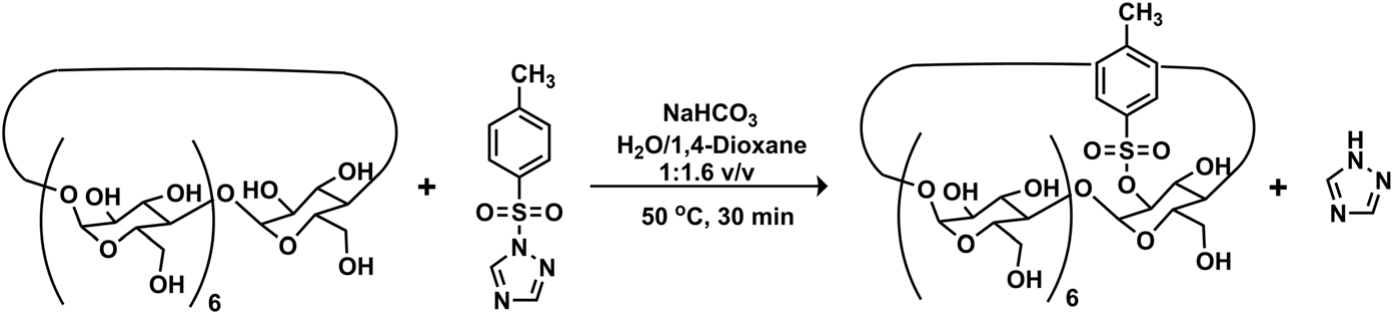
Reaction of β-CD with Ts-1*H*-Trz for the synthesis of 2^A^-*O*-Ts-β-CD under aqueous conditions.

In this work, we report the synthesis of 2^A^-*O*-Ts-β-CD using Ts-1*H*-Trz as a mild and easily handled tosylating reagent under aqueous, slightly basic conditions (NaHCO_3_) in a 1,4-dioxane/water system (Scheme 1). The method affords a 43% yield, with a decrease in the relative ESI-MS signal of the corresponding by-product ions, exceeding the yields obtained by the best previously reported procedures, with the exception of highly specialized mechanochemical approaches. In addition, the present protocol offers improved safety and operational simplicity, as it avoids the use of flammable and highly reactive NaH, allows the direct use of commercially available hydrated β-CD, and does not require an inert atmosphere.

## Results and discussion

Previous studies^31,39,40^ on the synthesis of 2^A^-*O*-Ts-β-CD in aqueous media have reported yields ranging from 10% to 34%. These methods typically employed Na_2_CO_3_, either alone or as a buffer in combination with NaHCO_3_, as the base; DMF or 1,4-dioxane as co-solvents; and Ts-Cl, Ts-Im, or 3-nitrophenyl 4-toluenesulfonate as the tosylating reagent.

**Scheme 2:**
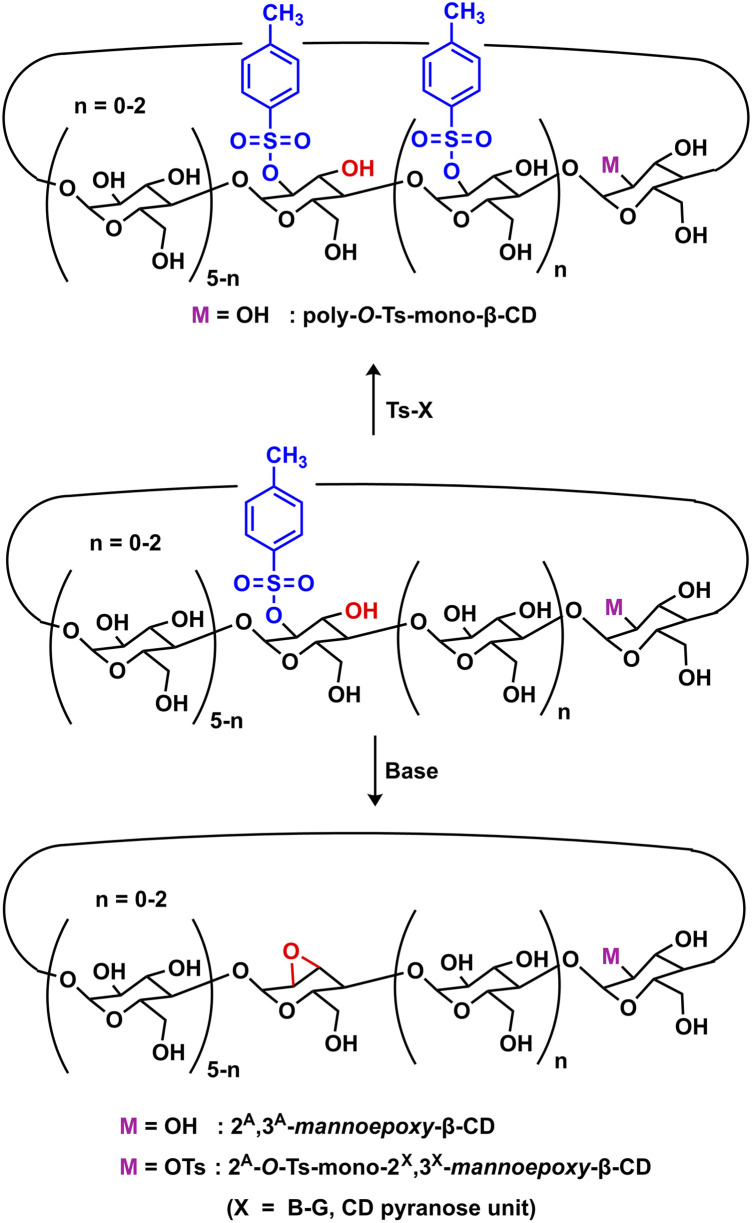
Polytosylated-, *mannoepoxy*- and *mannoepoxy*-tosylated-by-products formed *via* consecutive reactions of 2^A^-*O*-Ts-β-CD.

The synthesis of 2^A^-*O*-Ts-β-CD is complicated by the limited solubility of reactants and product and, more importantly, by selectivity issues in the tosylation reaction (mono/polytosylation and regioselectivity), as well as by side reactions including hydrolysis of the tosylating reagent and intramolecular epoxide formation (Scheme 2). All these factors reduce the yield of the desired mono-tosylated product and hinder its isolation and purification.

The presence of an organic co-solvent is essential to improve the solubility of both β-CD and the tosylating reagent. This is particularly important in mono-tosylation reactions, where complete dissolution of β-CD from the initial stages of the reaction is a prerequisite; otherwise, the formation of polytosylated derivatives is likely to occur. The effect of organic co-solvents on the solubility of β-CD in aqueous solutions has been investigated in the past.^43–46^ In multicomponent systems, however, the solubility behavior of β-CD is difficult to predict, as it is significantly influenced by a wide range of additives, including solvents, salts, ionic liquids, and related species.^47^

Thus, under typical tosylation conditions (*i.e.*, in the presence of a base and mild heating), the exact effect of organic solvents on the solubility of β-CD is not known. Four water-miscible, non-hydroxylic co-solvents (DMF, THF, 1,4-dioxane, DMSO) were evaluated based on two criteria: the minimum amount of organic solvent required to achieve complete dissolution of β-CD and the maximum amount tolerated before precipitation occurred. Among the solvents examined, DMF and 1,4-dioxane provided the broadest composition ranges over which β-CD remained fully soluble. Of these, 1,4-dioxane was selected because it exhibited slightly superior solubilizing performance and, owing to its lower boiling point, facilitated product isolation. Replacing Na_2_CO_3_ with NaHCO_3_ produced a similar solubility profile in water/1,4-dioxane mixtures.

**Fig. 1:**
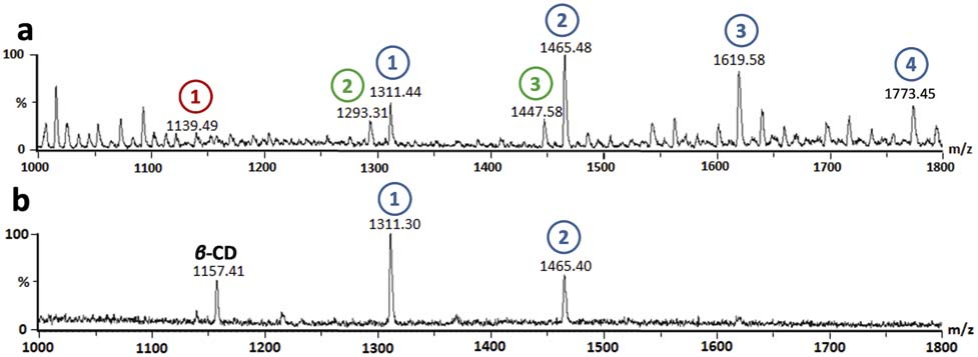
Expanded ESI-MS spectra (*m*/*z* 1000–1800) of reaction mixtures at the end of the reaction, showing the product distributions obtained under previously reported conditions^39^ (a) and the improved reaction conditions developed in the present study (b). Reaction conditions: (a) β-CD (1 equiv.) with Ts-Cl (3 equiv.), added portionwise over 1 h at 50 °C in H_2_O/1,4-dioxane (1 : 1, v/v) in the presence of Na_2_CO_3_ (3 equiv.), followed by an additional 30 min at 50 °C; (b) β-CD (1 equiv.) with Ts-1*H*-Trz (1.3 equiv.) in H_2_O/1,4-dioxane (1 : 1.6, v/v) in the presence of NaHCO_3_ (3 equiv.) for 30 min at 50 °C. β-CD: [β-CD + Na]^+^, 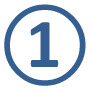: [2^A^-*O*-Ts-β-CD + Na]^+^, 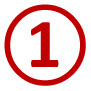: [2^A^,3^A^-*mannoepoxy*-β-CD + Na]^+^, 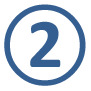: [di-2^A^,2^X^-*O*-Ts-β-CD + Na]^+^, 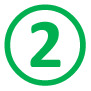: [2^A^-*O*-Ts-(2^X^,3^X^)-*mannoepoxy*-β-CD + Na]^+^, 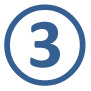: [tri-2^A^,2^X^,2^Y^-*O*-Ts-β-CD + Na]^+^, 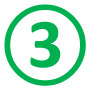: [di-2^A^,2^X^-*O*-Ts-(2^Y^,3^Y^)-*mannoepoxy*-β-CD + Na]^+^, 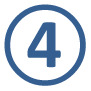: [tetra-2^A^,2^X^,2^Y^-2^Z^-*O*-Ts-β-CD + Na]^+^.

Initial experiments on the tosylation of β-CD were conducted using Ts-Cl as the tosylating reagent, 1,4-dioxane as a co-solvent, and NaOH, Na_2_CO_3_, NaHCO_3_, or Na_2_CO_3_/NaHCO_3_ mixtures as bases at room temperature, 40 °C, and 50 °C. Reaction progress was monitored by ESI-MS analysis of aliquots withdrawn at defined time intervals, following a methodology previously developed by our group.^42^ A major advantage of this approach is the straightforward identification of all reaction components, including unreacted β-CD, the desired monotosylated product, polytosylated derivatives, epoxides, and partially epoxidized polytosylates (Table S1). Although differences in ionization efficiency between structurally distinct species preclude reliable quantitative comparison of their abundances within a single spectrum, the ionization efficiency of a given compound is expected to remain essentially constant between samples analyzed under identical instrumental conditions. Consequently, comparisons of the intensity of the same ion across different spectra, as well as changes in product distributions under different reaction conditions, provide a reliable semi-quantitative measure of trends in product formation. Accordingly, all ESI-MS intensity comparisons presented in this work should be interpreted as semi-quantitative trends for individual species rather than as absolute measures of relative product amounts. Fig. 1 presents the results obtained using this methodology at the end of the reaction, comparing previously reported conditions,^39^ which afforded the desired product in 23% yield, with the reaction conditions identified in the present study, which increase the yield to 43%.

**Fig. 2:**
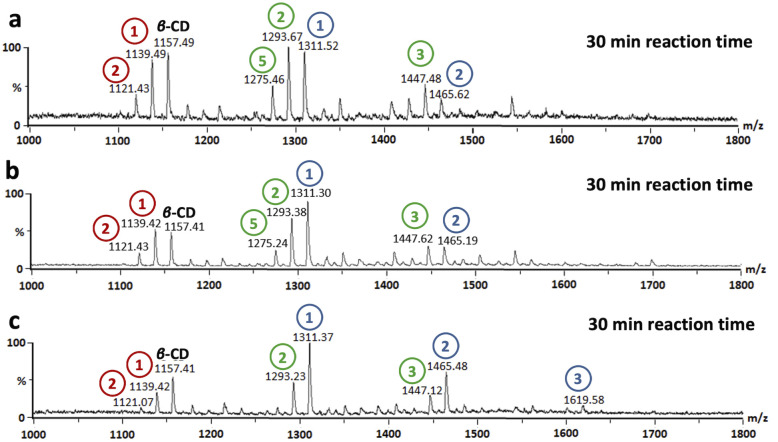
Effect of the H_2_O/1,4-dioxane ratio on product formation. Expanded ESI-MS spectra (*m*/*z* 1000–1800) of reaction mixtures obtained from β-CD (1.0 equiv.) and Ts-Cl (1.0 equiv.) in the presence of Na_2_CO_3_ (3.0 equiv.) at 50 °C for 30 min in H_2_O/1,4-dioxane at volume ratios of (a) 1 : 0.6; (b) 1 : 1.1 and (c) 1 : 1.6 (v/v). β-CD: [β-CD + Na]^+^, 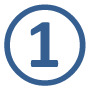: [2^A^-*O*-Ts-β-CD + Na]^+^, 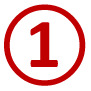: [2^A^,3^A^-*mannoepoxy*-β-CD + Na]^+^, 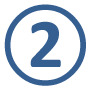: [di-2^A^,2^X^-*O*-Ts-β-CD + Na]^+^, 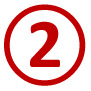: [di-(2^A^,3^A^),(2^X^,3^X^)-*mannoepoxy*-β-CD + Na]^+^, 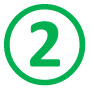: [2^A^-*O*-Ts-(2^X^,2^X^)-*mannoepoxy*-β-CD + Na]^+^, 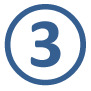: [tri-2^A^,2^X^,2^Y^-*O*-Ts-β-CD + Na]^+^, 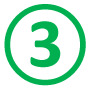: [di-2^A^,2^X^-*O*-Ts-(2^Y^,3^Y^)-*mannoepoxy*-β-CD + Na]^+^, 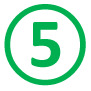: [2^A^-*O*-Ts-di-(2^X^,3^X^),(2^Y^,3^Y^)-*mannoepoxy*-β-CD + Na]^+^.

These experiments demonstrated that 50 °C is the optimal reaction temperature. Below this temperature, the solubility of β-CD was limited and the product yield decreased, whereas at higher temperatures significant conversion of the product to the corresponding epoxide was observed. This is consistent with the literature, since epoxide formation is typically achieved quantitatively using an aqueous NaHCO_3_ solution heated above 55 °C for several hours.^48,49^ Of particular importance was the observation that the reaction rate increased, while a decrease in the relative abundance of the *mannoepoxy*-related ESI-MS signals was observed as the 1,4-dioxane content in the aqueous medium was raised (Fig. 2), consistent with suppressed epoxide formation. Furthermore, the use of strong bases (Fig. S1 and S2, SI) and long reaction times also reduced the yield due to the formation of epoxidized tosylates and/or epoxides (Fig. S3, SI).

Based on the above results, the effects of the amounts of TsCl and Na_2_CO_3_ relative to β-CD were subsequently investigated. Since tosylation generates HCl as a by-product, requiring neutralization by the base to maintain a suitable reaction environment, TsCl and Na_2_CO_3_ were considered interdependent parameters and were evaluated together to identify efficient reaction conditions. The temperature was maintained at 50 °C, and the water/1,4-dioxane (v/v) ratio was fixed at 1 : 1.6, corresponding to the maximum value tolerated before precipitation occurred. As observed with Na_2_CO_3_ or the Na_2_CO_3_/NaHCO_3_ buffer system, the best results were obtained using equimolar amounts of base and Ts-Cl in the range of 1.2–1.8 equivalents (Fig. S4, SI) and a reaction time of 15 min (Fig. S5, SI). However, significant amounts of polytosylates and epoxidized tosylates were still formed, complicating product isolation. In contrast, when NaHCO_3_ was employed as the base, the corresponding ESI-MS signals for both polytosylates and epoxidized tosylates were generally less intense, although longer reaction times were required under these conditions.

Finally, the effect of the nature of the tosylating reagent was examined by replacing Ts-Cl with either Ts-Im or Ts-1*H*-Trz, using NaHCO_3_ as the base, which produces the lowest amount of by-products. Ts-Cl is highly reactive, whereas Ts-Im is considerably less reactive and Ts-1*H*-Trz displays intermediate reactivity.^42^ In a previous study,^42^ we found that, under anhydrous conditions (DMF, NaH), tosylation using nine different tosylating reagents afforded a narrow range of yields (33–40)%, with Ts-1*H*-Trz providing the highest yield. Although the reaction conditions employed in the present study differ substantially due to the use of aqueous media, it was nevertheless of interest to evaluate these alternative reagents.

With Ts-Cl, the yield reached up to 36%, although higher relative ESI-MS signals for by-product species were observed (Fig. S6, SI). On the other hand, reactions with the least reactive reagent Ts-Im proceeded slowly, requiring more than 6 h; consequently, significant amounts of epoxide by-products were also formed (Fig. S7, SI). Ts-1*H*-Trz showed the highest efficiency, affording an isolated yield of 43%, with the di-tosylated derivative as the only detectable by-product. Product formation reached its maximum after 30 min, while prolonged reaction times led to increased formation of the di-tosylated species, and tri-tosylated products also began to appear (Fig. S8, SI).

Tosylation at the 2- or 6-position, as a result of complexation of the tosylating reagent and its orientation within the cyclodextrin cavity, has occasionally been mentioned in the literature,^50–53^ particularly in aqueous solutions, where cyclodextrin encapsulation of guest molecules is considerably more pronounced.^52,54–57^

However, the data reported so far indicate that the contribution of this process is not always clear and may vary depending on the system under investigation.^33,58^ For example, in addition to the nature of the tosylating reagent, other factors, such as the presence of additional components in the reaction medium or an organic co-solvent, can strongly influence this process.

With this in mind, we aimed to examine the importance of this factor under the reaction conditions identified in the present study. Experiments were performed both in pure aqueous solution and under conditions resembling those of the reaction. Therefore, the complexation behavior of 1,4-dioxane, 1,2,4-triazole (Trz), sodium tosylate (TsONa) (formed as a side product during the reaction), and Ts-1*H*-Trz with β-CD was initially investigated in D_2_O by ^1^H NMR spectroscopy. The concentration was set at 2 mM, rather than the ∼34 mM used in the reaction, due to the limited solubility of some compounds in D_2_O. In the case of the Ts-1*H*-Trz/β-CD mixture, mild heating was required prior to NMR data acquisition to prevent precipitation of the β-CD complex.

**Fig. 3:**
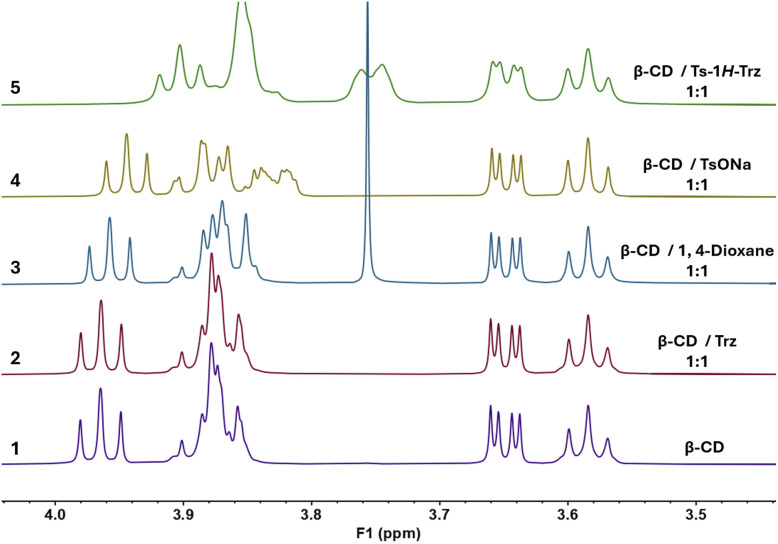
Expanded ^1^H NMR spectra in the β-CD proton region in D_2_O (600 MHz, 25 °C). Spectra were recorded for (1) β-CD (2 mM) and equimolar mixtures (2 mM each) of β-CD with (2) Trz, (3) 1,4-dioxane, (4) TsONa, and (5) Ts-1*H*-Trz.

Chemical shift changes of the CD-H3 and CD-H5 protons, located within the interior of the cyclodextrin cavity, were used to identify complex formation.^59–61^ The results presented in Fig. 3 indicate that, although Trz does not form a detectable complex at a concentration of 2 mM, all other compounds undergo complexation to varying extents, following the order of complexation efficiency: Ts-1*H*-Trz ≫ TsONa > 1,4-dioxane.

The above experiments were also repeated in the presence of a small amount (5% v/v) of DMSO-*d*_6_, where no solubility issues were present under these conditions. Although DMSO-*d*_6_ at 5% v/v was found to form a complex with β-CD, the conclusions regarding the relative complexation efficiencies of Ts-1*H*-Trz, 1,4-dioxane, and TsONa remained unchanged (Fig. S9, SI).

For 1,4-dioxane, as shown in Fig. S10, maximum complexation is achieved only at β-CD/1,4-dioxane molar ratios greater than 1 : 10 (β-CD concentration = 10 mM in D_2_O).

The strong complexation observed between Ts-1*H*-Trz and β-CD suggests that tosylation of β-CD at the 2-position by Ts-1*H*-Trz may indeed proceed *via* the formation of a host–guest complex, as previously proposed in the literature for other tosylating systems.^31^ However, as already mentioned, the complexation process depends not only on the reactant itself, but also on its concentration, as well as on the presence and concentrations of all other components in the reaction medium. Accordingly, to verify that this conclusion remains valid under the actual reaction conditions, the complexation behavior was investigated in the presence of all relevant components of the reaction medium, including NaDCO_3_ and a large excess of 1,4-dioxane.

**Fig. 4:**
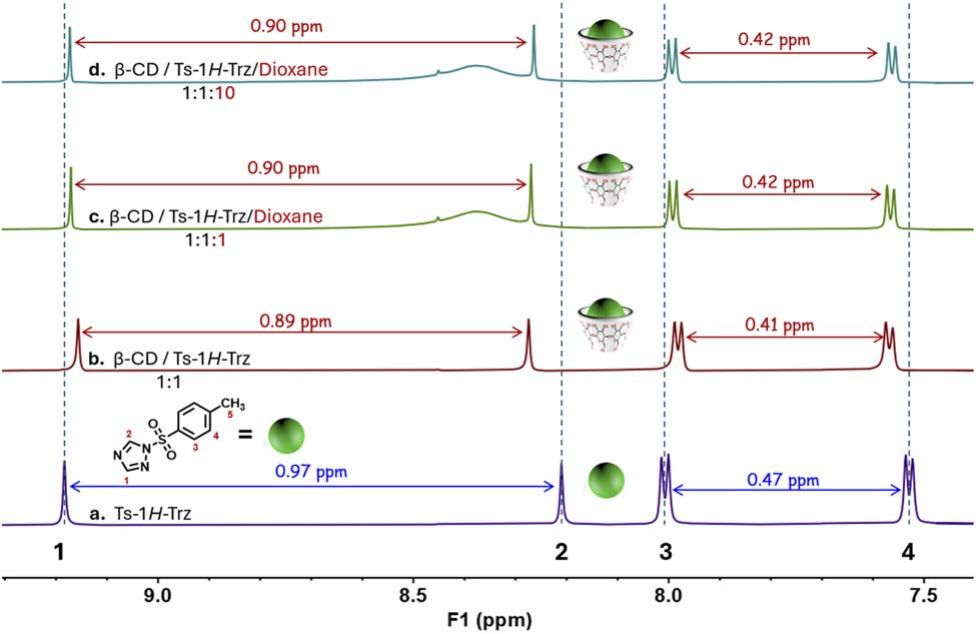
Expanded ^1^H NMR spectra in the β-CD proton region in D_2_O (600 MHz, 25 °C): (a) Ts-1*H*-Trz (2 mM); (b) β-CD/Ts-1*H*-Trz (2 mM : 2 mM); (c) β-CD/Ts-1*H*-Trz/1,4-dioxane (2 mM : 2 mM : 2 mM); and (d) β-CD/Ts-1*H*-Trz/1,4-dioxane (2 mM : 2 mM : 20 mM).

Nevertheless, the presence of 1,4-dioxane at high concentration represents a significant complication, as its intense ^1^H NMR resonance can overlap with and mask the signals of interest. Although the use of deuterated 1,4-dioxane would largely eliminate this issue, it was not employed due to its high cost. Therefore, in this case the complexation process was investigated by monitoring changes in the chemical shifts of the guest molecule (Ts-1*H*-Trz) in the aromatic region, which are free from interference by the 1,4-dioxane signal. The chemical shift changes associated with complexation of Ts-1*H*-Trz by β-CD are relatively small, yet significant. Thus, as shown in Fig. 4, the chemical shifts observed for the singlets at 9.18 ppm and 8.02 ppm and the doublets at 8.21 ppm and 7.55 ppm differ between free and complexed Ts-1*H*-Trz.

Since the reaction proceeds rapidly at temperatures close to the reaction temperature identified in the present study (50 °C), typically within minutes, studies under these conditions proved difficult. Consequently, the experiments were performed at room temperature; however, at this temperature the limited solubility of β-CD and its complexes introduced an additional complication.

Given this limitation, which precluded the use of conditions identical to those present at the initial stage of the reaction, it was considered that the major factors governing competitive complexation of β-CD with either 1,4-dioxane or Ts-1*H*-Trz were the 1,4-dioxane to water ratio and the 1,4-dioxane to Ts-1*H*-Trz molar ratio. Therefore, three sets of experiments were performed using a fixed D_2_O/1,4-dioxane volume ratio of 1 : 1.6 (v/v), consistent with the reaction conditions. In the first set, the concentrations of β-CD and Ts-1*H*-Trz were 5.1 mM and 6.6 mM, respectively, representing the final stages of the reaction, where the concentrations of both reactants are expected to be substantially reduced. In contrast, in the other two sets, the β-CD concentration was fixed at 15.2 mM, while the Ts-1*H*-Trz concentration was set at 19.8 mM and 44.0 mM, respectively, to more closely represent the intermediate and initial stages of the reaction (Table 1).

**Table 1:** Conditions employed in three ^1^H NMR experiments to evaluate inclusion complex formation between β-CD and Ts-1*H*-Trz. The reaction conditions identified in the present study for the synthesis of 2^A^-*O*-Ts-β-cyclodextrin are included for comparison

Set of experiment	[β-CD] (mmol L^−1^)	[Ts-1*H*-Trz] (mmol L^−1^)	Molar ratio (1,4-dioxane/Ts-1*H*-Trz)	Molar ratio (Ts-1*H*-Trz/β-CD)	Molar ratio (NaDCO_3_/β-CD)
1	5.1	6.6	1086.3	1.3	3.0
2	15.2	19.8	363.7	1.3	3.0
3	15.2	44.0	163.4	2.9	3.0
Reaction	33.9	44.0	163.5	1.3	3.0
Conditions					

**Fig. 5:**
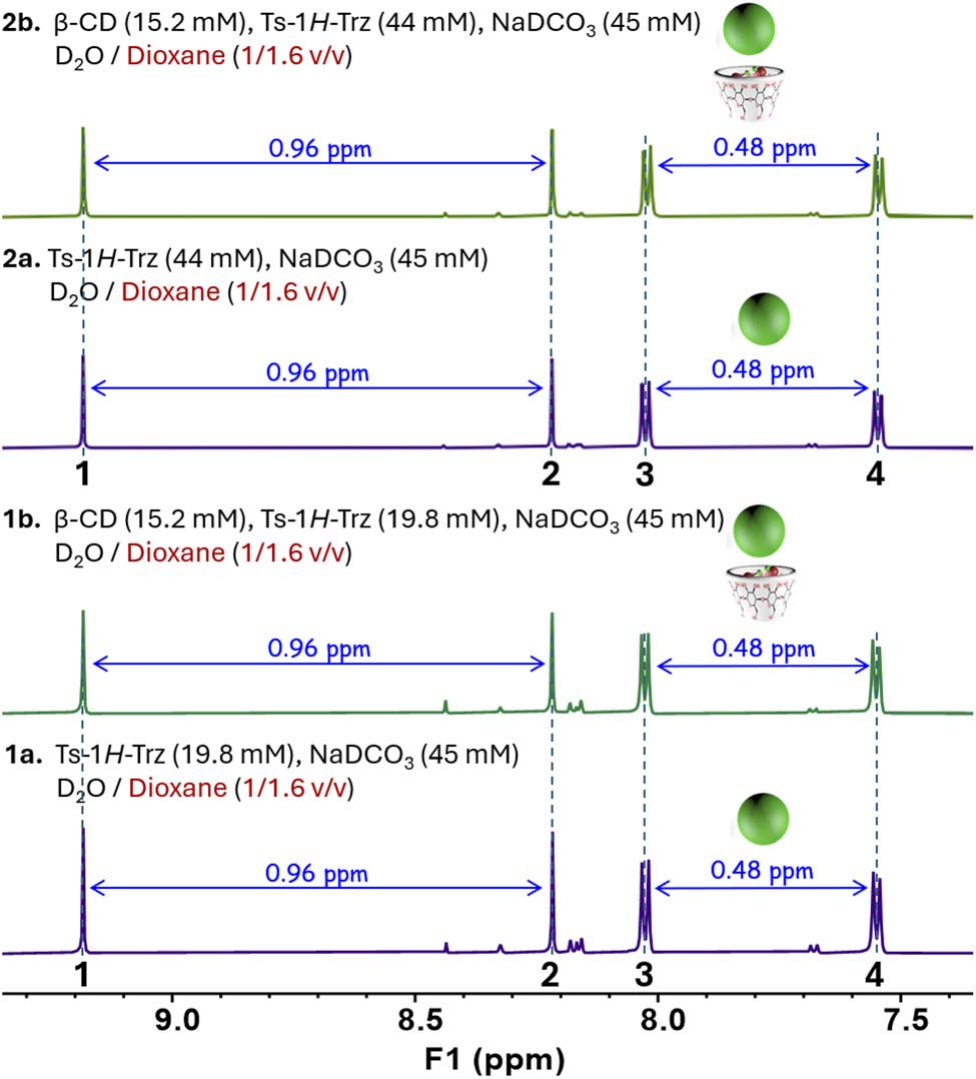
Expanded view of the ^1^H NMR (600 MHz, 25 °C) spectra in the aromatic region in D_2_O/1,4-dioxane (500 µL, 1 : 1.6 v/v): (1a) Ts-1*H*-Trz (19.8 mM), NaDCO_3_ (45 mM); (1b) β-CD (15.2 mM), Ts-1*H*-Trz (19.8 mM), NaDCO_3_ (45 mM); (2a) Ts-1*H*-Trz (44.0 mM), NaDCO_3_ (45 mM); (2b) β-CD (15.2 mM), Ts-1*H*-Trz (44.0 mM), NaDCO_3_ (45 mM).

Under all three experimental conditions, no NMR evidence for significant inclusion of Ts-1*H*-Trz within the β-CD cavity was observed in the presence of a large excess of 1,4-dioxane (Fig. 5). To further examine this point two 2D ROESY experiments were performed. One experiment was carried out in a 30% CD_3_CN in D_2_O solvent system, where host–guest complex formation is expected to occur, while the second was performed in the D_2_O/1,4-dioxane solvent mixture at the same 1 : 1.6 (v/v) ratio used in the reaction. Acetonitrile was used to increase the solubility of both β-CD and Ts-1*H*-Trz and, at the same time, served as a weakly bound guest.^62^

As expected, in the CD_3_CN/D_2_O solvent system, ROESY cross-peaks were observed between the β-CD cavity protons (H-3 and H-5) and the aromatic protons of Ts-1*H*-Trz, confirming host–guest interactions. In contrast, no corresponding cross-peaks were detected in the D_2_O/1,4-dioxane solvent system, indicating that a high 1,4-dioxane/D_2_O ratio does not favor β-CD/Ts-1*H*-Trz complexation (Fig. S11, SI). Consistent with the ROESY results, the presence of β-CD induced ^1^H NMR chemical-shift changes in the aromatic protons of Ts-1*H*-Trz only in the CH_3_CN/H_2_O solvent system, whereas no such changes were observed in the D_2_O/1,4-dioxane reaction medium.

Although the exact reaction conditions, particularly those prevailing during the very initial stage of the reaction and at the reaction temperature, could not be fully reproduced, the present findings indicate that, under the experimental conditions examined, no detectable β-CD/Ts-1*H*-Trz inclusion complex was observed in the presence of a high 1,4-dioxane/D_2_O ratio. These results suggest that stable inclusion complexes are not formed under these conditions. However, they do not exclude the possibility that transient or low-population β-CD·Ts-1*H*-Trz complexes may form during the reaction. Consequently, the available NMR data neither provide evidence for nor conclusively rule out the involvement of such species in the reaction mechanism.

## Experimental

### Materials and methods

Reagents were obtained from Sigma-Aldrich, Merck, TCI Europe or Fluka and were used without further purification while solvents were obtained from Merck and Honeywell. Commercially available hydrated β-cyclodextrin (calculated to 88.8% β-cyclodextrin) was used without any treatment. 1-Tosyl-(1*H*)-imidazole (Ts-Im) and 1-tosyl-(1*H*)-1,2,4-triazole (Ts-1*H*-Trz) were prepared according to the procedure described in our previous work.^42^ Sodium deuterobicarbonate (NaDCO_3_) was prepared by dissolving 100 mg NaHCO_3_ in 2 mL D_2_O, followed by solvent removal under reduced pressure. This dissolution/evaporation cycle was repeated three times to ensure complete H/D exchange, and the resulting solid was dried under high vacuum (oil pump) to constant weight prior to use. 1,4-Dioxane was distilled over LiAlH_4_ in inert atmosphere (Ar) and stored in inert atmosphere (Ar) over 4A molecular sieves. Merck silica gel 60 F_254_ plates 0.25 mm were used for TLC whereas, and visualized using UV light (254 nm) or staining with charring method using 35% sulfuric acid (H_2_SO_4_) spray and then heating with heat gun. Reverse column chromatography was performed with RP-silica gel Merck LiChroprep RP-18 (40–63 µm). High-resolution mass spectra (HRMS) was acquired using an H-ESI Orbitrap Exploris 120 mass spectrometer (Thermo Scientific). Electrospray ionization mass spectrometry (ESI-MS) experiments were performed with a Waters MicroMass ZQ Electrospray Platform coupled to a MassLynx V4.1 data system (Waters GmbH, Vienna, Austria). NMR spectra were recorded on a Bruker Avance II spectrometer at 600 MHz for ^1^H NMR and 151 MHz for ^13^C NMR, using D_2_O or DMSO-*d*_6_ as solvents. Ultra-precision 5 mm NMR tubes purchased from Norell. Chemical shifts are reported (multiplicity, coupling constant, integration) on the *δ* scale in parts per million (ppm) downfield from tetramethylsilane (*δ* = 0.0 ppm) using the residual solvent signal at *δ* = 4.79 ppm (^1^H NMR, D_2_O), *δ* = 2.50 ppm (^1^H NMR, DMSO-*d*_6_), *δ* = 39.52 ppm (^13^C NMR, DMSO-*d*_6_) as internal standard. The following abbreviations were used in reporting NMR data: s = singlet, d = doublet, t = triplet, m = multiplet. The 2D ROESY spectra were acquired with a spectral width of 7211.5 Hz (F2) and 7204.6 Hz (F1), 1024 data points in t2, 64 scans, 403 complex points in t1, and a relaxation delay of 2.0 s. The mixing time in the 2D ROESY experiment was 250 ms.

### Synthetic procedures

#### General procedure followed in β-cyclodextrin solubility experiments in the presence or absence of base or salt

Hydrated β-CD (28 mg, 2.48 × 10^−5^ mol), with or without a base/basic salt (0–5.0 equiv. relative to β-CD), was suspended in H_2_O (0.2 mL) at the target temperature. The organic co-solvent was added dropwise until the cloudy suspension cleared, and addition continued until turbidity reappeared, giving the minimum solvent volume required for complete dissolution and the maximum tolerated before precipitation.

#### General procedure used for the evaluation of reaction parameters in the synthesis of 2A-*O*-Ts-β-cyclodextrin

Hydrated β-CD (112.6 mg, 0.099 mmol) and the appropriate base or basic salt (1–3 equivalents relative to β-CD) were suspended in 1 mL of water and heated to the target temperature, resulting in a cloudy solution. 1,4-Dioxane was then added to achieve the desired H_2_O/1,4-dioxane volume ratio, affording a clear solution. Subsequently, the tosylating reagent (0.5–3 equivalents relative to β-CD) was added, and the reaction mixture was stirred at the same temperature. At predetermined time intervals, aliquots were withdrawn and analyzed by mass spectrometry. ESI-MS samples were prepared by diluting an aliquot of the reaction mixture (10 µL) with H_2_O/iPrOH (3 : 1, 500 µL) containing NaCl (0.2 M, 20 µL). The reaction time varied according to the results of the mass spectrometric analysis. Selected reactions were further processed as follows. The reaction mixture was quenched with 1 M HCl (pH = 5–6) and concentrated under reduced pressure.

##### Case A (Ts-Cl as the tosylating reagent)

The reaction mixture was dissolved in H_2_O/CH_3_CN (9 : 1, *ca.* 1 mL). Trace insoluble material (<3 mg), identified as a mixture of polytosylated β-CD by-products (representative case by ESI-MS, Fig. S12, SI), when present, was removed by centrifugation prior to loading onto the column. The solution was loaded onto an RP-18 silica gel column (*ø* 15 × 120 mm) and eluted initially with 1.2 CV H_2_O/CH_3_CN (9 : 1), followed by 2CV H_2_O/CH_3_CN (8 : 2). Fractions containing the product, 2^A^-*O*-Ts-β-CD (eluted with H_2_O/CH_3_CN, 8 : 2), were combined and concentrated under reduced pressure to afford the product as a white solid.

##### Case B (Ts-1*H*-Trz and Ts-Im as tosylating reagents)

To remove non-polar components that can introduce problems during column chromatography, the resulting white solid was dissolved in DMF (∼0.5 mL), and acetone (2 mL) was added dropwise, resulting in the formation of a white precipitate. Et_2_O (8 mL) was then added portionwise to complete precipitation. The precipitate was collected by centrifugation and washed with Et_2_O/acetone (4 : 1, 6 mL), followed by acetone (5 mL), affording a white solid. The crude product was dried and dissolved in H_2_O/CH_3_CN (9 : 1, *ca.* 1 mL). Trace insoluble material (<3 mg), identified as a mixture of polytosylated β-CD by-products (representative case by ESI-MS, Fig. S12, SI), when present, was removed by centrifugation prior to loading onto the column. The solution was loaded onto an RP-18 silica gel column (*ø* 15 × 120 mm) and eluted initially with 1.2 CV H_2_O/CH_3_CN (9 : 1), followed by 2 CV H_2_O/CH_3_CN (8 : 2). Fractions containing the product, 2^A^-*O*-Ts-β-CD (eluted with H_2_O/CH_3_CN, 8 : 2), were combined and concentrated under reduced pressure to afford the product as a white solid.

#### Procedure for the synthesis of 2^A^-*O*-Ts-β-cyclodextrin with Ts-1*H*-Trz

In hydrated β-cyclodextrin (337.4 mg, 0.264 mmol) and NaHCO_3_ (66.5 mg, 0.792 mmol) were added 3 mL of H_2_O and heated to 50 °C, affording a cloudy solution. Upon addition of 4.8 mL of 1,4-dioxane (resulting in a 1.6 : 1 ratio of 1,4-dioxane to H_2_O), the solution became clear, and Ts-1*H*-Trz (76.6 mg, 0.343 mmol) was added, and the reaction mixture was stirred at 50 °C for 30 min. The reaction was quenched with 1 M HCl (343 µL, 0.343 mmol) and evaporated *in vacuo*. To remove non-polar components that can introduce problems during column chromatography, the resulting white solid was dissolved in 1.5 mL of DMF, followed by a dropwise addition of 6 mL acetone, which produced a white precipitate. Subsequently, 24 mL of Et_2_O were added portion wise to complete precipitation. The precipitate was separated by centrifugation and washed with 20 mL of Et_2_O/acetone (4 : 1), followed by 14 mL of acetone, affording a white solid. The crude was dried and dissolved in approximately 1.5 mL of H_2_O/CH_3_CN (9 : 1). Trace insoluble material (<3 mg), identified as a mixture of polytosylated β-CD by-products (representative case by ESI-MS, Fig. S12, SI), when present, was removed by centrifugation prior to loading onto the column. The solution was loaded on RP-18 silica gel column (*ø* 25 × 120 mm) and eluted initially with 1.2 CV H_2_O/CH_3_CN (9 : 1) and followed by 2 CV H_2_O/CH_3_CN (8 : 2). Evaporation of the fractions containing the product, 2^A^-*O*-Ts-β-CD (eluted with H_2_O/CH_3_CN, 8 : 2), gave a white solid (146.1, 43%). *R*_f_ = 0.77 (iPrOH/H_2_O 7 : 3); ^1^H NMR (600 MHz; D_2_O; Fig. S13, SI) *δ* ppm: 7.97 (d, ^3^*J*_*bc*_ = 8.1 Hz, 2H; H_*b*_), 7.54 (d, ^3^*J*_*bc*_ = 8.1 Hz, 2H; H_*c*_), 5.14–5.07 (m, 6H; H-1), 4.88 (s overlapped with HDO signal, 1H; H-1′), 4.43 (broad s, 1H; H-2′), 4.19 (apparent t, H-3′), 4.01–3.56 (m, 39H; H-2 H-3 H-4 H-5 H-6), 2.49 (s, 3H; H_*e*_); ^1^H NMR (600 MHz; DMSO-*d*_6_; Fig. S14, SI) *δ* ppm: 7.85 (d, ^3^*J*_*bc*_ = 8.1 Hz, 2H; H_*b*_), 7.44 (d, ^3^*J*_*bc*_ = 8.1 Hz, 2H; H_*c*_), 5.93–5.48 (m, 13H; OH-2 OH-3), 4.87–4.80 (m, 7H; H-1 H-1′), 4.50–4.29 (m, 7H; OH-6), 4.15 (apparent d, ^3^*J*_23_ = 3.6 Hz, 1H; H-2′), 4.06 (dd, ^3^*J*_34_ = 10.0 Hz, ^3^*J*_23_ = 3.6 Hz, 1H; H-3′), 3.91 (apparent td, 1H; H-5′), 3.80–3.20 (m overlapped with H_2_O signal, 39H; H-2 H-3 H-4 H-5 H-6), 2.42 (s, 3H; H_*e*_); ^13^C NMR (151 MHz; DMSO-*d*_6_; Fig. S15, SI) *δ* ppm: 144.8 (C_*a*_), 132.9 (C_*d*_), 129.8 (C_*c*_), 128.0 (C_*b*_), [102.0, 101.9, 101.8, 101.7 (C-1)], 98.1 (C-1′), [81.6, 81.6, 81.5, 81.4 (C-1)], 80.9 (C-4′), 79.6 (C-2′), [73.1, 73.0, 72.9, 72.8, 72.4, 72.4, 72.3, 72.1, 72.0, 72.0, 71.9, 71.9, 71.6 (C-2, C-3, C-5)], 69.3 (C-3′), [59.9, 59.8, 59.7 (C-6, C-6′)], 21.1 (C_*e*_); HRMS (ESI) (Fig. S16, SI) *m*/*z*: calcd for C_49_H_76_NaO_37_S 1311.3684, found 1311.3696 [M + Na]^+^.

## Conclusions

In this work, an improved and efficient protocol for the synthesis of 2^A^-*O*-Ts-β-CD was developed under aqueous, mildly basic conditions. The study demonstrates that careful control of solvent composition, temperature, and base strength is essential to balance β-cyclodextrin solubility with suppression of undesired side reactions, including polytosylation and epoxide formation. A 1,4-dioxane/water system in combination with NaHCO_3_ proved optimal, with 50 °C identified as the most suitable reaction temperature.

Among the investigated tosylating reagents, Ts-1*H*-Trz showed the best overall performance, providing the highest isolated yield (43%) and cleaner reaction profiles compared to Ts-Cl and Ts-Im.

The developed protocol provides a safer and operationally straightforward approach, offering improved practicality using commercially available hydrated β-cyclodextrin and avoiding strictly anhydrous conditions, while affording higher yields compared with previously reported aqueous methods. Also, it provides a practical and efficient route to 2^A^-*O*-Ts-β-cyclodextrin, an important synthetic intermediate that enables the preparation of a broad range of regioselectively functionalized cyclodextrin derivatives. By simplifying access to this key precursor under operationally straightforward conditions, the method is expected to facilitate future developments in cyclodextrin functionalization and the preparation of advanced supramolecular systems. Although NMR measurements could not be performed under conditions fully identical to those of the reaction, particularly during its initial stages and at the reaction temperature, the data obtained under conditions closely resembling different stages of the reaction provided no evidence for the formation of a detectable β-CD/Ts-1*H*-Trz inclusion complex. However, the involvement of transient or low-population β-CD/Ts-1*H*-Trz complexes during the reaction cannot be conclusively ruled out.

## Author contributions

Conceptualization, G. G. K., G. M. T.; data curation: G. G. K., G. M. T.; formal analysis, G. G. K., G. M. T.; investigation, M. T., G. G. K.; methodology, G. G. K., G. M. T.; project administration: G. G. K., G. M. T.; resources, G. M. T.; supervision: G. G. K., G. M. T.; visualization: M. T., G. G. K., G. M. T.; writing – original draft: G. G. K., G. M. T.; writing – review and editing: G. G. K., G. M. T. All authors have read and agreed to the published version of the manuscript.

## Conflicts of interest

There are no conflicts to declare.

## Supplementary Material

RA-OLF-D6RA05416A-s001

## Data Availability

The data supporting this article have been included as part of the supplementary information (SI). Supplementary information is available. See DOI: https://doi.org/10.1039/d6ra05416a.
