# Supplementary material for: Easily accessible 2A-O-Ts-β-cyclodextrin via efficient aqueous conditions
Source: RSC Adv. 2026 Aug 17;16(41):48339–47. doi: 10.1039/d6ra05416a (PMC13479953; doi:10.1039/d6ra05416a)
Supplement: Supplementary file 1 [file RA-016-D6RA05416A-s001.pdf]

Supplementary Information (SI) for RSC Advances.
This journal is © The Royal Society of Chemistry 2026

Supporting Information

Easily Accessible 2^A-*O*-Ts-β-Cyclodextrin *via* Efficient Aqueous Conditions

Golfo G. Kordopati,* Maria Tsouna, and Gerasimos M. Tsivgoulis*

Department of Chemistry, University of Patras, 26504, Greece

Corresponding authors:
G. G. Kordopati (gkordopati@upatras.gr)
G. M. Tsivgoulis (g.tsivgoulis@upatras.gr)

Data For supporting information

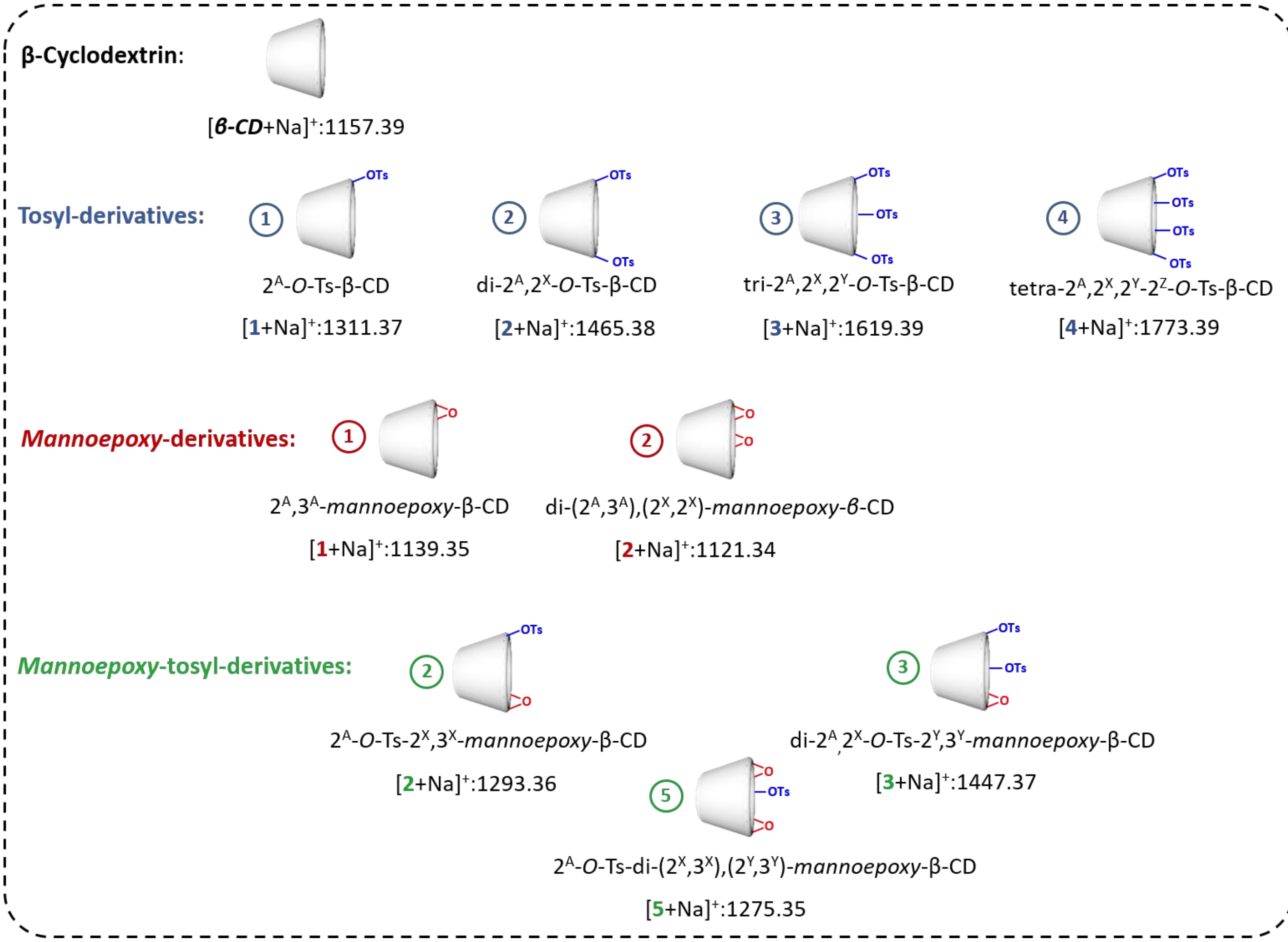

Scheme S1. Structures of cyclodextrin derivatives identified by ESI-MS analysis, along with their corresponding compound names and exact masses.

Table S1. Summary of the investigated reaction conditions and the corresponding outcomes during the evaluation of the aqueous synthesis of 2^{A}-*O*-Ts-β-CD. β-CD (1 equiv.) was used as the limiting reagent in all reactions. All reactions listed in this table were conducted at 50 °C.

Entry	Ts-X (equiv)	Base (equiv)	H_2O/dioxane (v/v)	Optimal Reaction Time	Yield* (isolated)	Normalized ESI-MS relative intensities# (β-CD peak = 100%)
**H_2O/1,4-dioxane ratio (TsCl fixed at 1.0 equiv., Na_2CO_3 3.0 equiv.)**						
1	TsCl (1.0)	Na_2CO_3 (3.0)	1:0.6	30 min	-	①: 102%, ②: 30%, ①: 91%, ②: 39%, ②: 109%, ③: 52%, ⑤: 52%
2	TsCl (1.0)	Na_2CO_3 (3.0)	1:1.1	30 min	-	①: 191%, ②: 55%, ①: 118%, ②: 36%, ②: 146%, ③: 55%, ⑤: 46%
3	TsCl (1.0)	Na_2CO_3 (3.0)	1:1.6	30 min	-	①: 200%, ②: 114%, ③: 17%, ①: 58%, ②: 9%, ②: 92%, ③: 50%
**Excess of (TsCl / Na_2CO_3)**						
4	TsCl (1.2)	Na_2CO_3 (1.2)	1:1.6	30 min	**34%**	-
5	TsCl (1.4)	Na_2CO_3 (1.4)	1:1.6	30 min	**35%**	-
6	TsCl (1.6)	Na_2CO_3 (1.6)	1:1.6	15 min	**36%**	-
7	TsCl (1.8)	Na_2CO_3 (1.8)	1:1.6	15 min	**36%**	-
8	TsCl (2.5)	Na_2CO_3 (2.5)	1:1.6	15 min	**32%**	-
9	TsCl (3.0)	Na_2CO_3 (3.0)	1:1.6	15 min	**29%**	-
**Base identity / strength (TsCl fixed at 1.0 equiv.)**						
10	TsCl (1.0)	Na_2CO_3 (3.0)	1:1.6	30 min	-	①: 180%, ②: 100%, ③: 10%, ①: 50%, ②: 10%, ②: 80%, ③: 45%
11	TsCl (1.0)	Na_2CO_3 (1.5)	1:1.6	30 min	-	**Optimal Reaction Time**<br>**30 min:** ①: 94%, ②: 56%, ③: 11%, ①: 33%, ②: 25%, ③: 10%<br>**Additional Information**<br>**15 min:** ①: 141%, ②: 104%, ③: 22%, ①: 22%, ②: 22%<br>**3h:** ①: 150%, ②: 92%, ③: 17%, ①: 42%, ②: 8%, ②: 75%, ③: 42%, ⑤: 8%
12	TsCl (1.0)	Na_2CO_3 (1.0) / $NaHCO_3$ (2.0)	1:1.6	30 min	-	①: 79%
13	TsCl (1.0)	$NaHCO_3$ (1.5)	1:1.6	30 min	-	①: 92%, ②: 30%, ①: 27%
**Ts-Im / $NaHCO_3$**						
14	Ts-Im (1.2)	$NaHCO_3$ (1.2)	1:1.6	18 h	-	**Optimal Reaction Time**<br>**18h:** ①: 109%, ②: 36%, ①: 49%, ②: 30%<br>**Additional Information**<br>**3h:** ①: 16%<br>**6h:** ①: 90%, ①: 11%

Table S1. (continued)

**Ts-1*H*-Trz equiv screening ($NaHCO_3$ fixed at 2.0 equiv)**						
15	Ts-1*H*-Trz (1.0)	$NaHCO_3$ (2.0)	1:1.6	30 min	**36%**	-
16	Ts-1*H*-Trz (1.1)	$NaHCO_3$ (2.0)	1:1.6	30 min	**37%**	-
17	Ts-1*H*-Trz (1.2)	$NaHCO_3$ (2.0)	1:1.6	30 min	**38%**	-
18	Ts-1*H*-Trz (1.3)	$NaHCO_3$ (2.0)	1:1.6	30 min	**39%**	-
19	Ts-1*H*-Trz (1.4)	$NaHCO_3$ (2.0)	1:1.6	30 min	**39%**	-
**$NaHCO_3$ equiv screening (Ts-1*H*-Trz fixed at 1.3 equiv)**						
20	Ts-1*H*-Trz (1.3)	$NaHCO_3$ (1.5)	1:1.6	30 min	**36%**	-
21	Ts-1*H*-Trz (1.3)	$NaHCO_3$ (2.5)	1:1.6	30 min	**40%**	-
22	Ts-1*H*-Trz (1.3)	$NaHCO_3$ (3.0)	1:1.6	30 min	**43%**	**Optimal Reaction Time**<br>**30 min:** ①**:** 211%, ②**:** 111%<br><br>**Additional Information:**<br>**1h:** ①**:** 238%, ②**:** 188%, ③**:** 31%, ①: 38%, ②: 37%<br>**2h:** ①**:** 271%, ②**:** 200%, ③**:** 42%, ①: 43%, ②: 47%
23	Ts-1*H*-Trz (1.3)	$NaHCO_3$ (4.0)	1:1.6	30 min	**43%**	-

* *Entries marked with "–" correspond to reaction conditions evaluated by comparative ESI-MS screening only.*

\# *Peak intensities were normalized to the unreacted β-CD peak (set to 100%) within each individual ESI-MS spectrum. Values represent relative abundance within a given spectrum and do not reflect absolute quantification, given potential differences in ionization efficiency between species. Comparisons of the same species across different entries in this table (e.g., as a function of reaction time or conditions) are considered semi-quantitative, whereas comparisons between different species within the same spectrum are not.* ①: 2^{A}-*O*-Ts-β-CD, ②: di-2^{A},2^{X}-*O*-Ts-β-CD, ③: tri-2^{A},2^{X},2^{Y}-*O*-Ts-β-CD, ①: 2^{A},3^{A}-*mannoepoxy*-β-CD, ②: 2^{A}-*O*-Ts-2^{X},3^{X}-*mannoepoxy*-β-CD, ②: 2^{A}-*O*-Ts-2^{X},3^{X}-*mannoepoxy*-β-CD , ③: di-2^{A},2^{X}-*O*-Ts-2^{Y},3^{Y}-*mannoepoxy*-β-CD, ⑤: [2^{A}-*O*-Ts-di-(2^{X},3^{X}),(2^{Y},3^{Y})-*mannoepoxy*-β-CD.

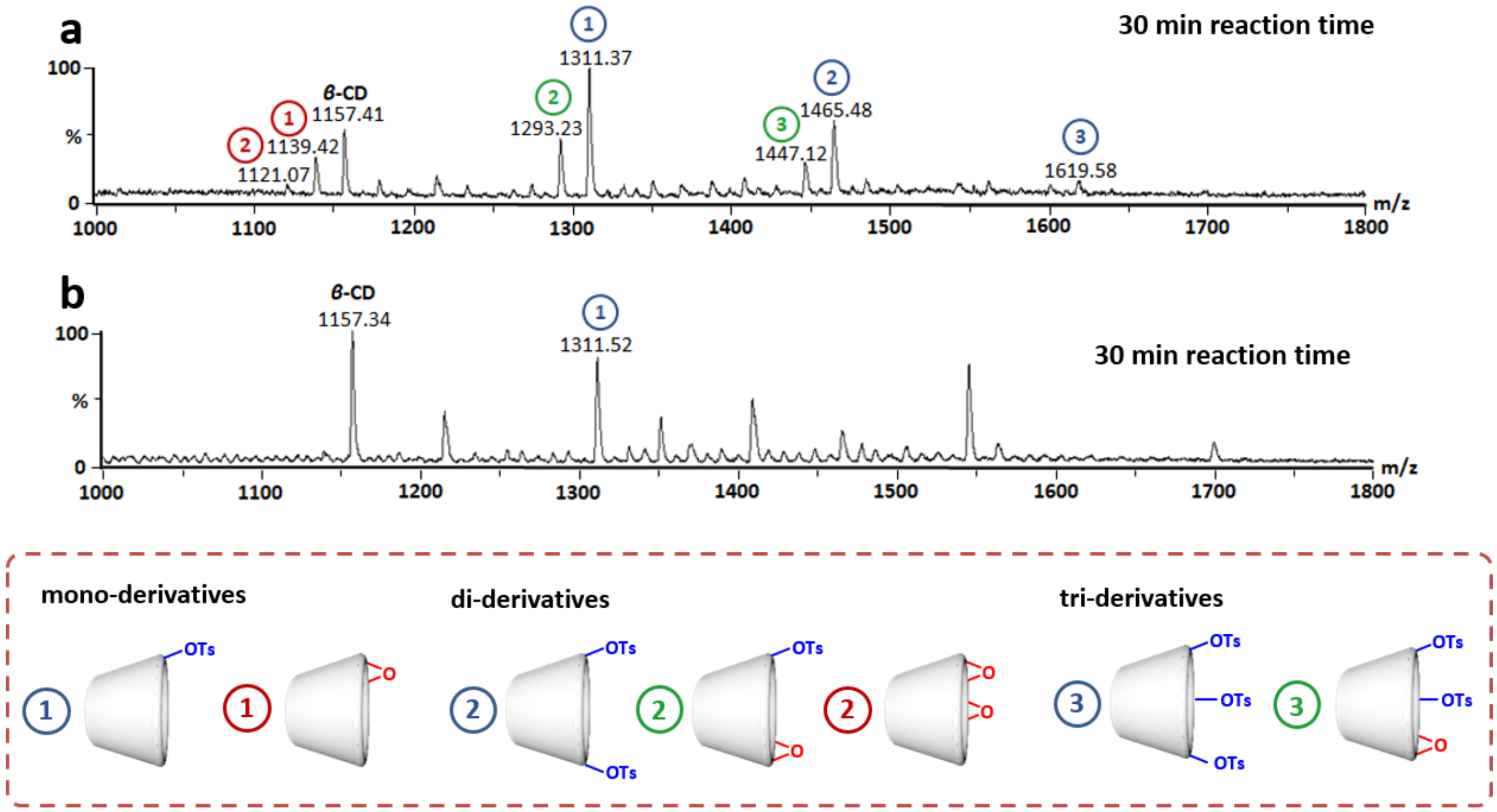

Fig. S1 Comparison between Na_2CO_3 and $Na_2CO_3/NaHCO_3$ as base. Expanded ESI–MS spectra (m/z 1000–1800) of reaction aliquots collected at the end of the reaction of β-CD (1.0 equiv), Ts-Cl (1.0 equiv) in H_2O/1,4-dioxane (1:1.6, v/v) at 50 °C for 30 min in the presence of: (**a**) Na_2CO_3 (3 equiv) and (**b**) Na_2CO_3 (1 equiv) / $NaHCO_3$ (2.0 equiv).

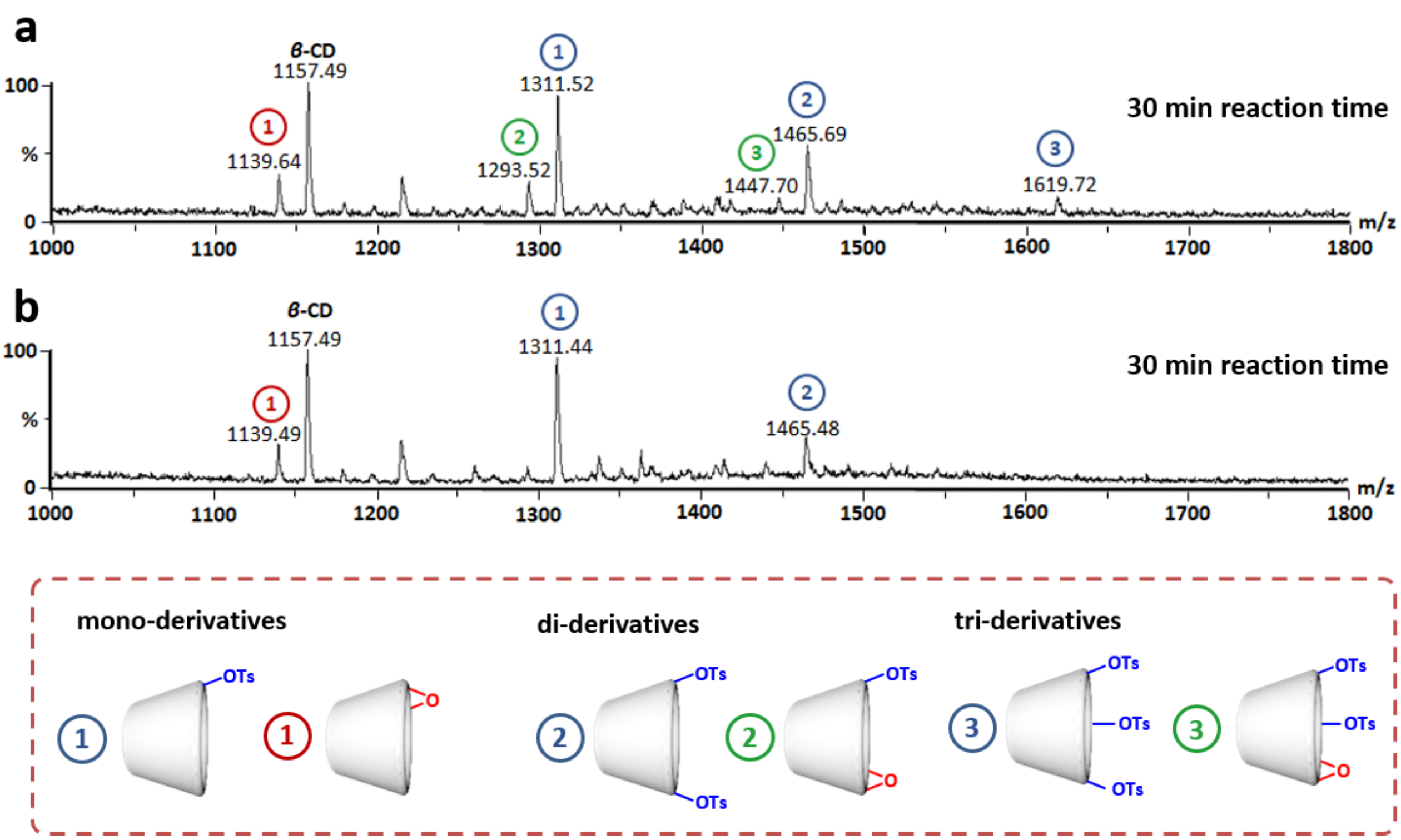

Fig. S2 Comparing the effect of base strength on product distribution. Expanded ESI–MS spectra (m/z 1000–1800) of reaction aliquots collected at the end of the reaction of β-CD (1.0 equiv), Ts-Cl (1.0 equiv) in H_2O/1,4-dioxane (1:1.6, v/v) at 50 °C for 30 min in the presence of: (**a**) Na_2CO_3 (1.5 equiv) and (**b**) $NaHCO_3$ (1.5 equiv).

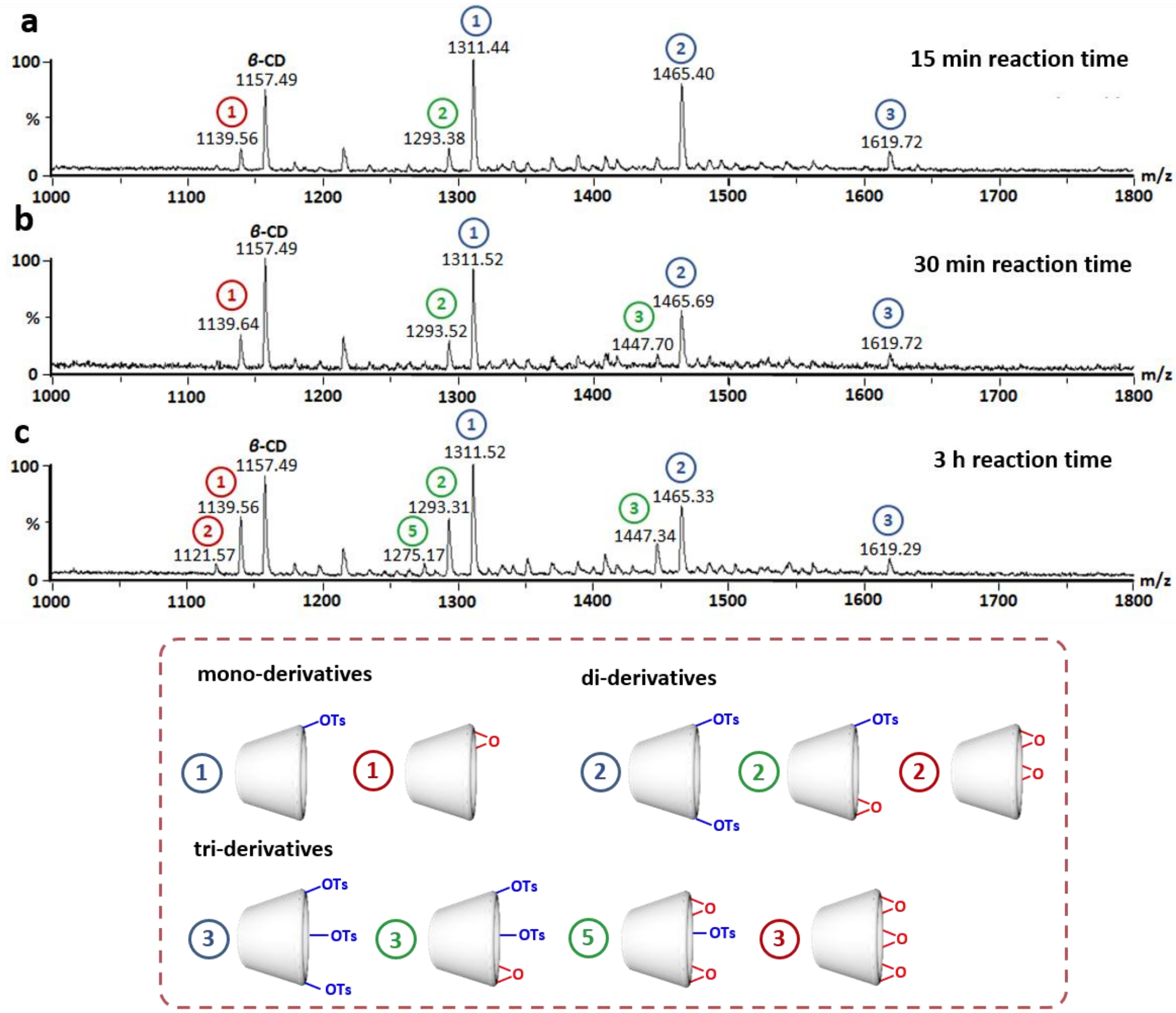

Fig. S3 Effect of reaction time on product formation with Na_2CO_3 as the base. Expanded ESI–MS spectra (m/z 1000–1800) of reaction aliquots collected during the reaction of β-CD (1.0 equiv) with Ts-Cl (1.0 equiv) in H_2O/1,4-dioxane (1:1.6, v/v) using Na_2CO_3 (1.5 equiv) as the base at 50 °C. Spectra were recorded after reaction times of (**a**) 15 min, (**b**) 30 min, and (**c**) 3 h.

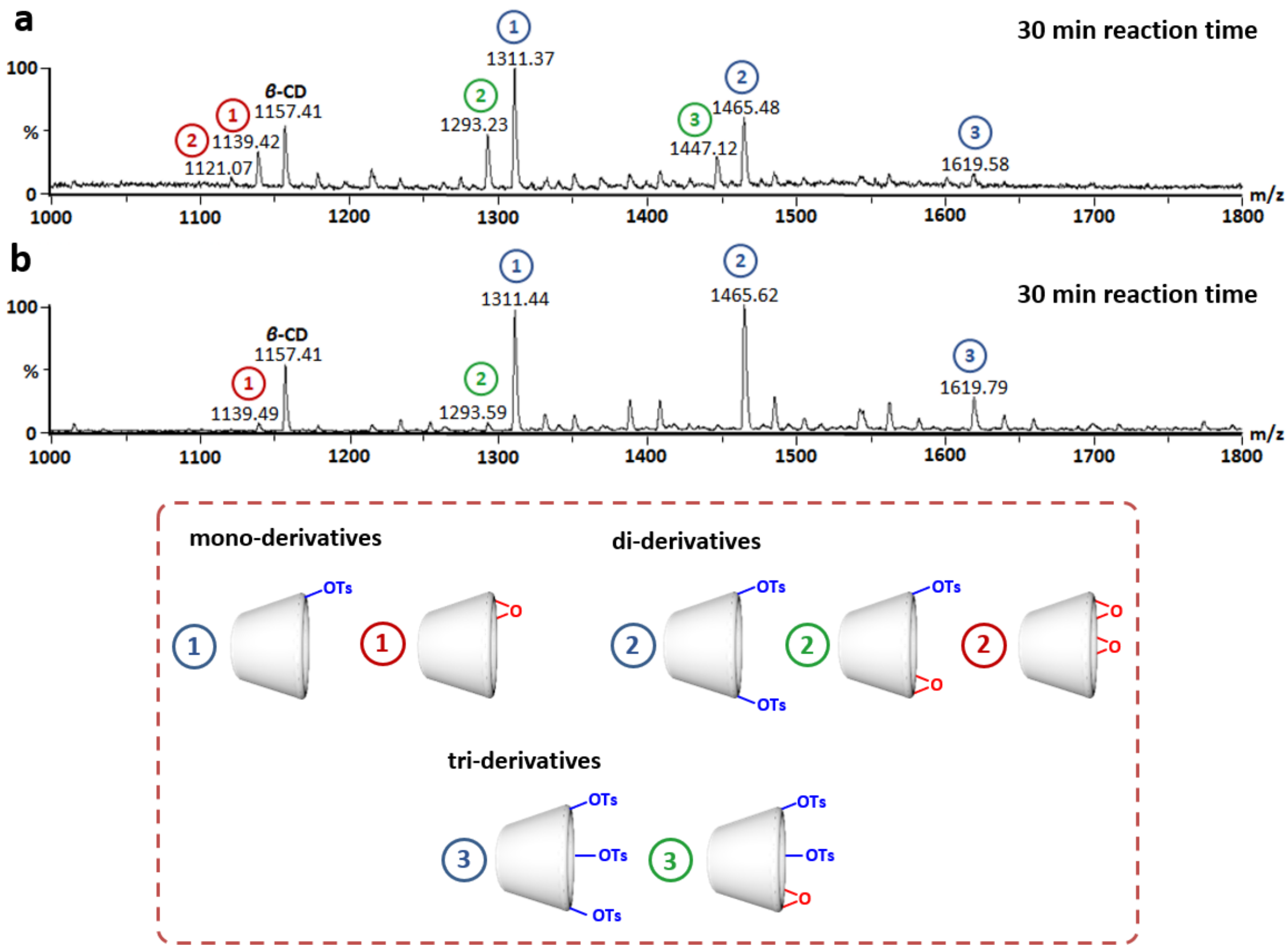

Fig. S4 Effect of Na_2CO_3 and Ts-Cl stoichiometry on product formation. Expanded ESI–MS spectra (m/z 1000–1800) of reaction mixtures obtained from β-CD (1 equiv) in H_2O/1,4-dioxane (1:1.6, v/v) at 50 °C for 30 min using (**a**) Ts-Cl (1 equiv) and Na_2CO_3 (3 equiv), and (**b**) Ts-Cl (1.2 equiv) and Na_2CO_3 (1.2 equiv).

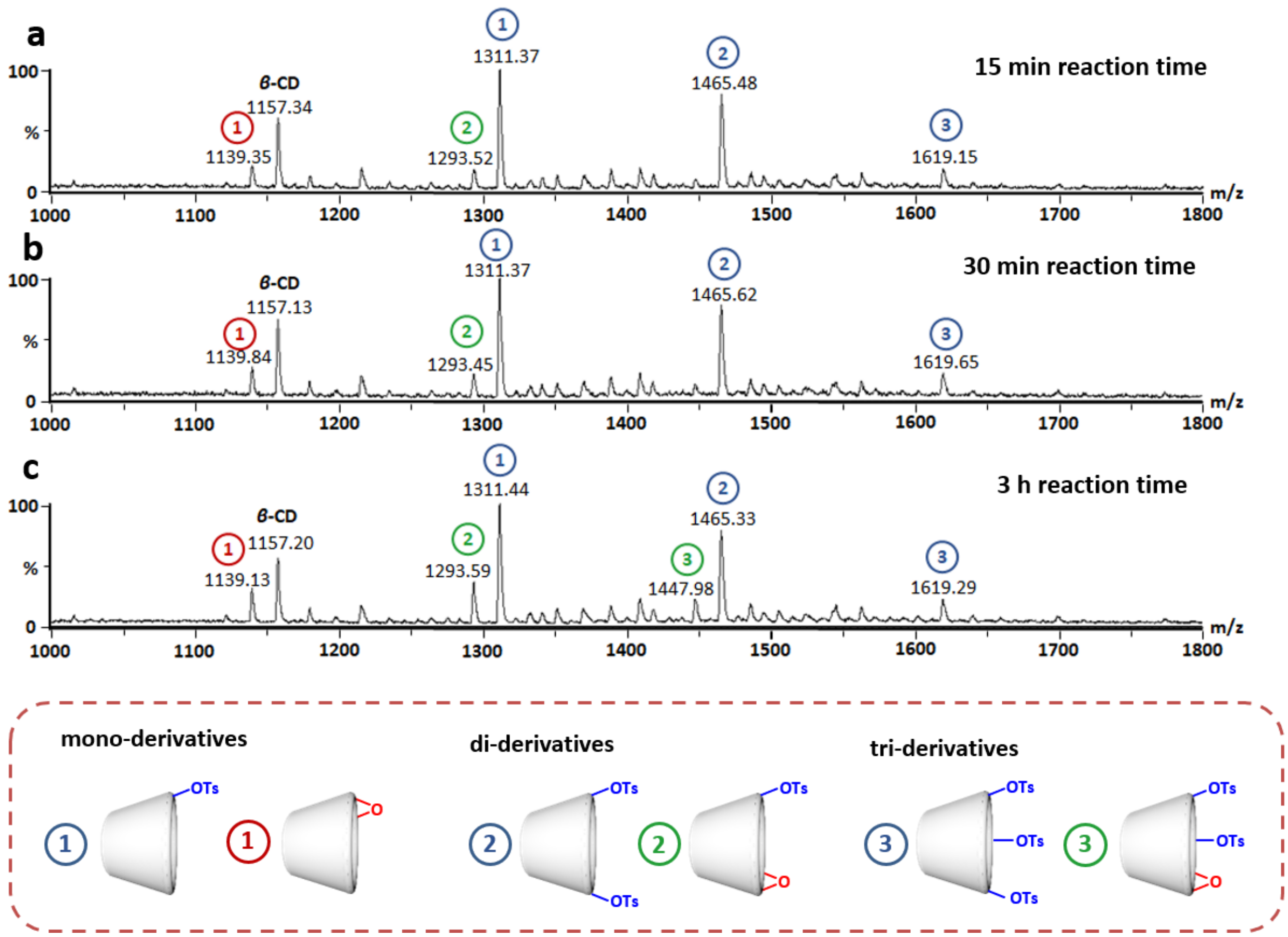

Fig. S5 Effect of reaction time on product formation using equimolar amounts of Na_2CO_3, $NaHCO_3$ and Ts-Cl. Expanded ESI–MS spectra (m/z 1000–1800) of reaction aliquots collected during the reaction of β-CD (1.0 equiv) with Ts-Cl (1.2 equiv) in H_2O/1,4-dioxane (1:1.6, v/v) in the presence of Na_2CO_3 (1.2 equiv) and $NaHCO_3$ (1.2 equiv) at 50 °C. Spectra were recorded after reaction times of (**a**) 15 min, (**b**) 30 min, and (**c**) 3 h of reaction.

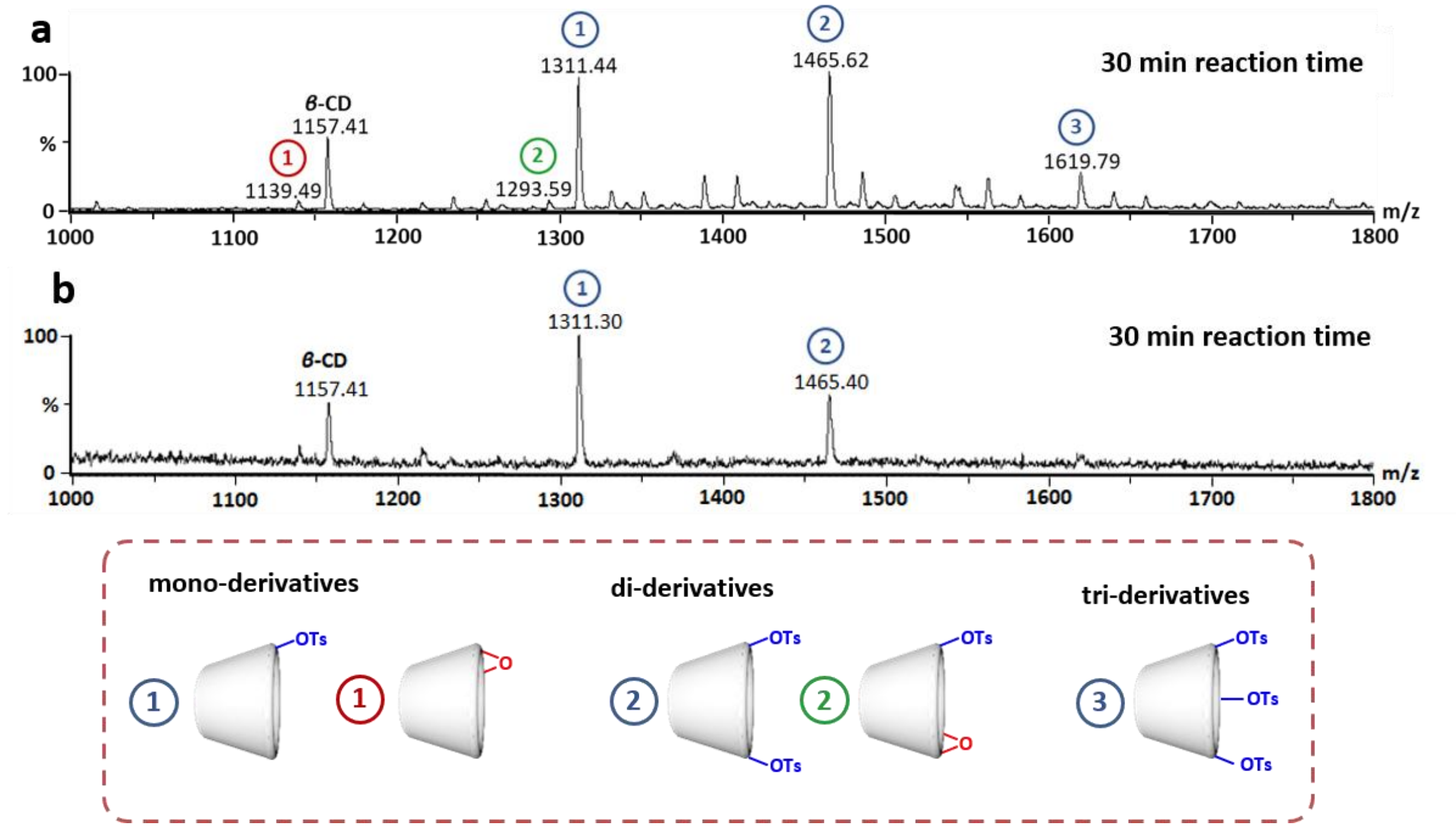

Fig. S6 Comparison of two tosylating systems: Ts-Cl/Na_2CO_3 and Ts-1*H*-Trz/$NaHCO_3$. Expanded ESI–MS spectra (m/z 1000–1800) of reaction aliquots collected at the end of the reaction of β-CD (1.0 equiv) in H_2O/1,4-dioxane (1:1.6, v/v) at 50 °C for 30 min under the following conditions: (**a**) Ts-Cl (1.2 equiv) in the presence of Na_2CO_3 (1.2 equiv) and (**b**) Ts-1*H*-Trz (1.3 equiv) in the presence of $NaHCO_3$ (3.0 equiv).

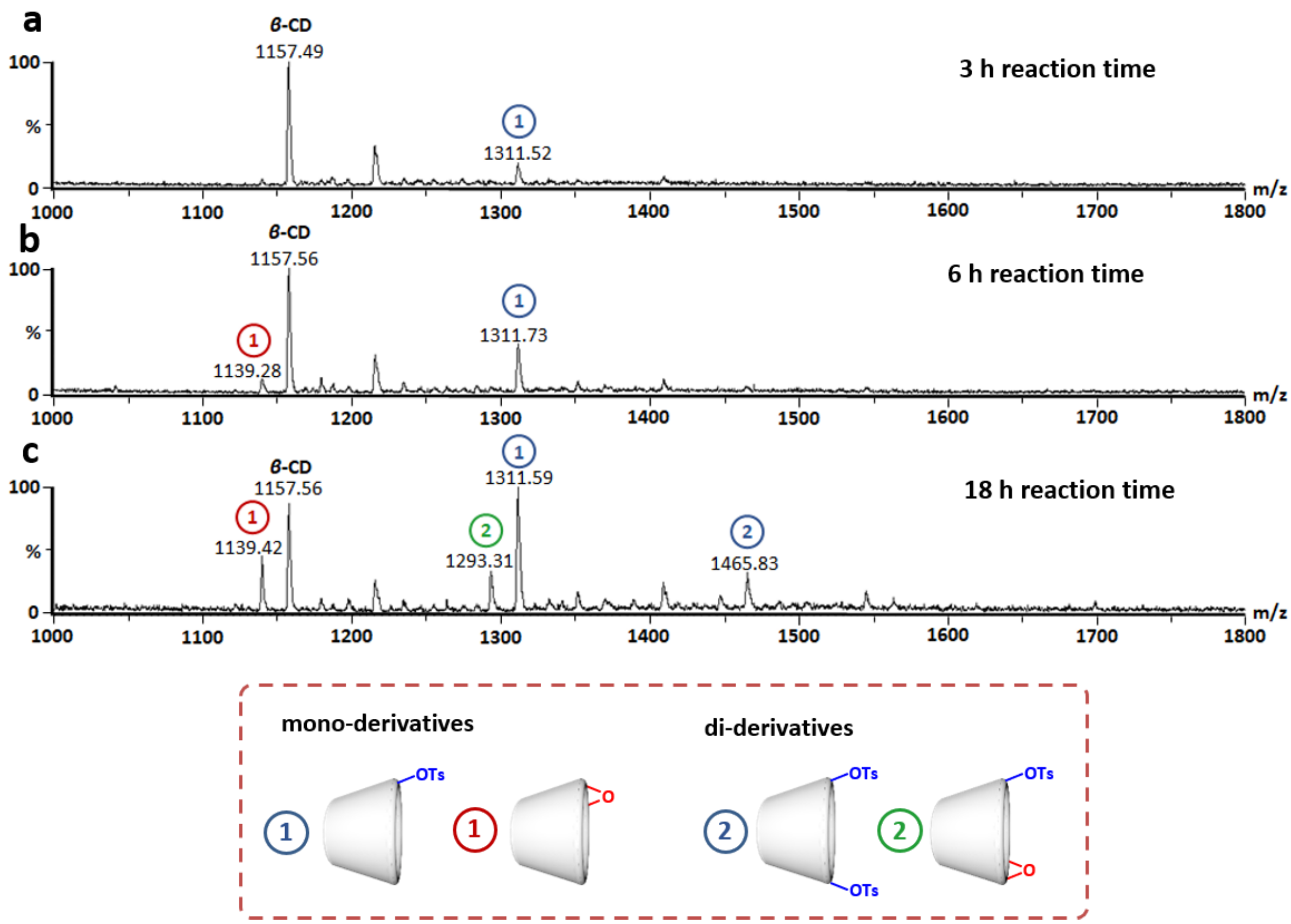

Fig. S7 Tosylation of β-CD using Ts-Im as the tosylating reagent. Expanded ESI–MS spectra (m/z 1000–1800) of reaction aliquots collected during the reaction of β-CD (1.0 equiv) with Ts-Im (1.2 equiv) in H_2O/1,4-dioxane (1:1.6, v/v) in the presence of $NaHCO_3$ (1.2 equiv) at 50 °C. Spectra were recorded after reaction times of (**a**) 3 h, (**b**) 6 h, and (**c**) 18 h.

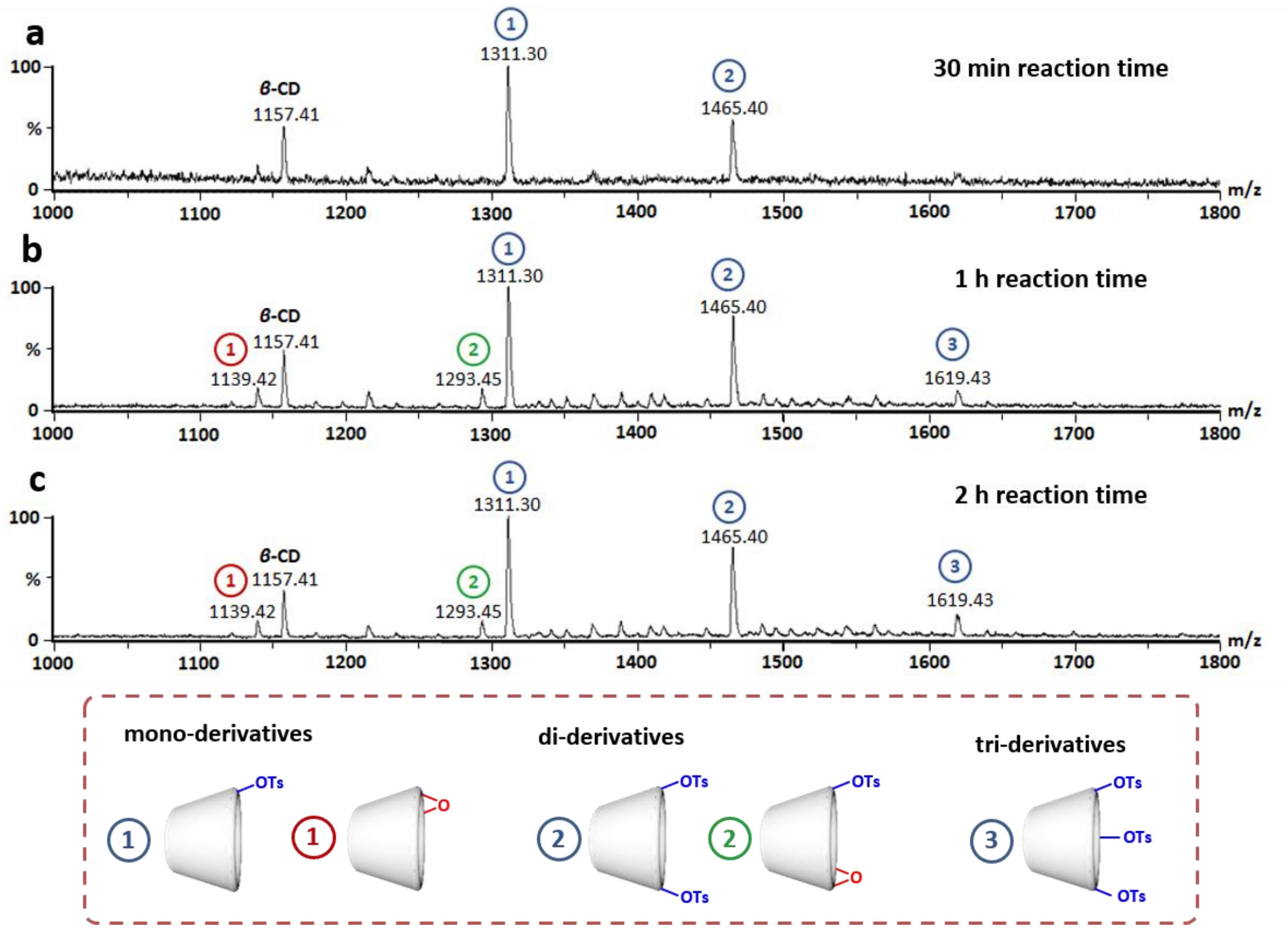

Fig. S8 Effect of reaction time on product formation using Ts-1*H*-Trz as the tosylating reagent and $NaHCO_3$ as the base under the reaction conditions identified in the present study. Expanded ESI–MS spectra (m/z 1000–1800) of reaction aliquots collected during the reaction of β-CD (1.0 equiv) with Ts-1*H*-Trz (1.3 equiv) in H_2O/1,4-dioxane (1:1.6, v/v) in the presence of $NaHCO_3$ (3.0 equiv) at 50 °C. Spectra were recorded after reaction times of (**a**) 30 min, (**b**) 1 h, and (**c**) 2 h.

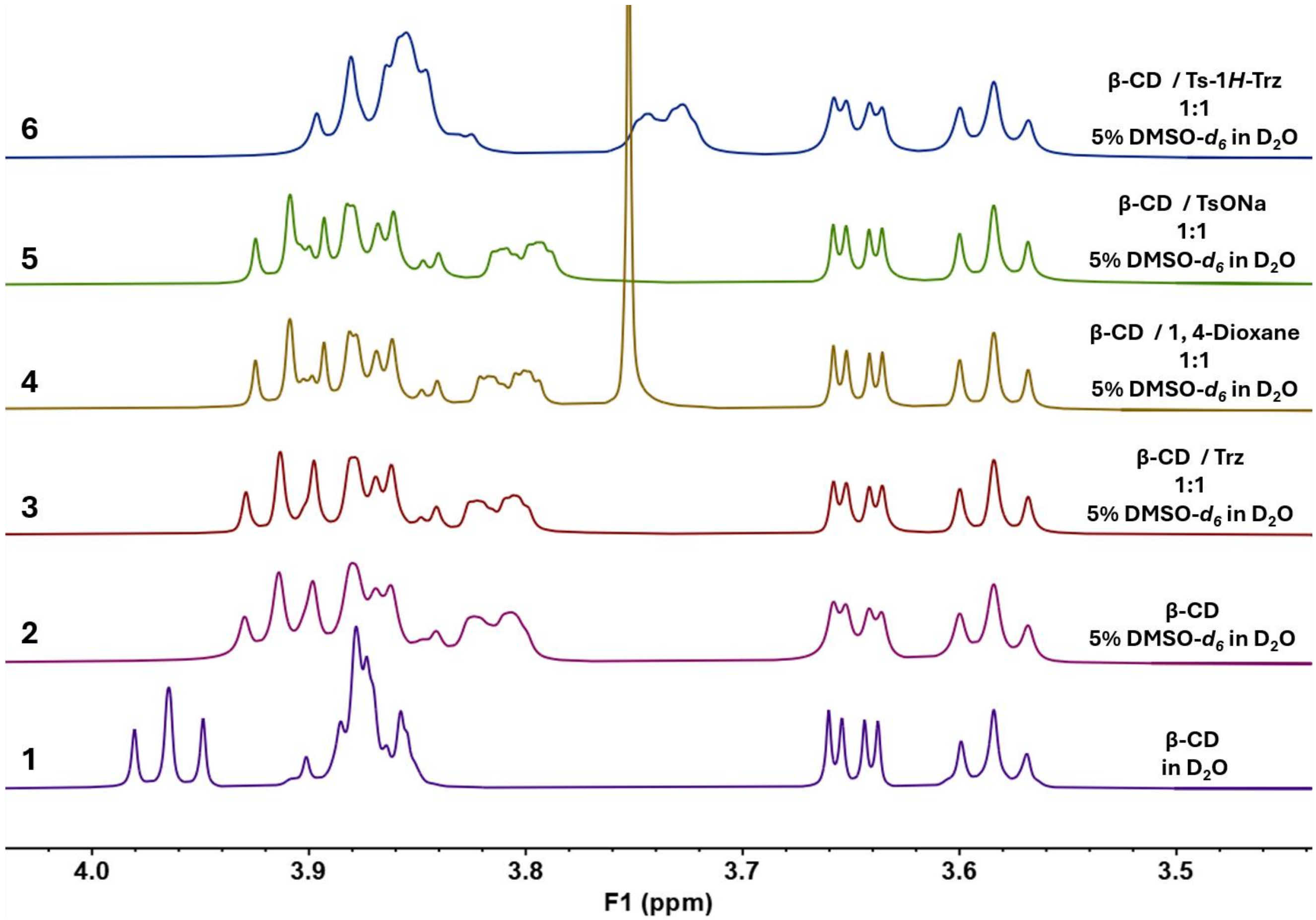

Fig. S9 Expanded ^1H NMR spectra in the β-CD proton region (600 MHz, 25 °C): (**1**) β-CD (2 mM) in D_2O; (**2**) β-CD (2 mM) in 5% DMSO-d_6/D_2O; and equimolar mixtures (2 mM each) of β-CD.

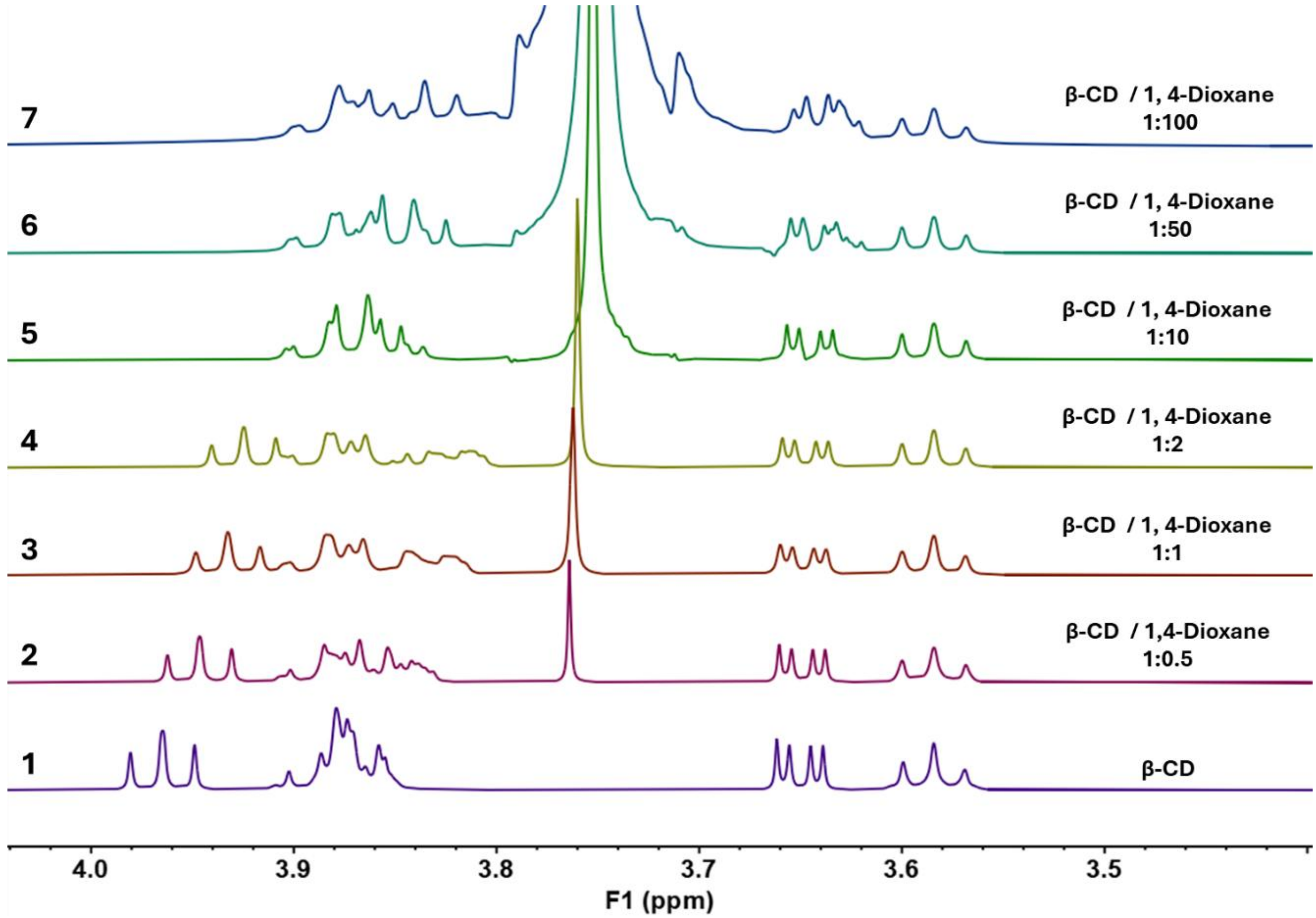

Fig. S10 Expanded ^1H NMR spectra in the β-CD proton region in D_2O (600 MHz, 25 °C). Spectra were recorded for (**1**) β-CD (10 mM) and β-CD/1,4-dioxane mixtures containing β-CD at 10 mM and 1,4-dioxane at (**2**) 5 mM, (**3**) 10 mM, (**4**) 20 mM, (**5**) 100 mM, (**6**) 500 mM, and (**7**) 1000 mM.

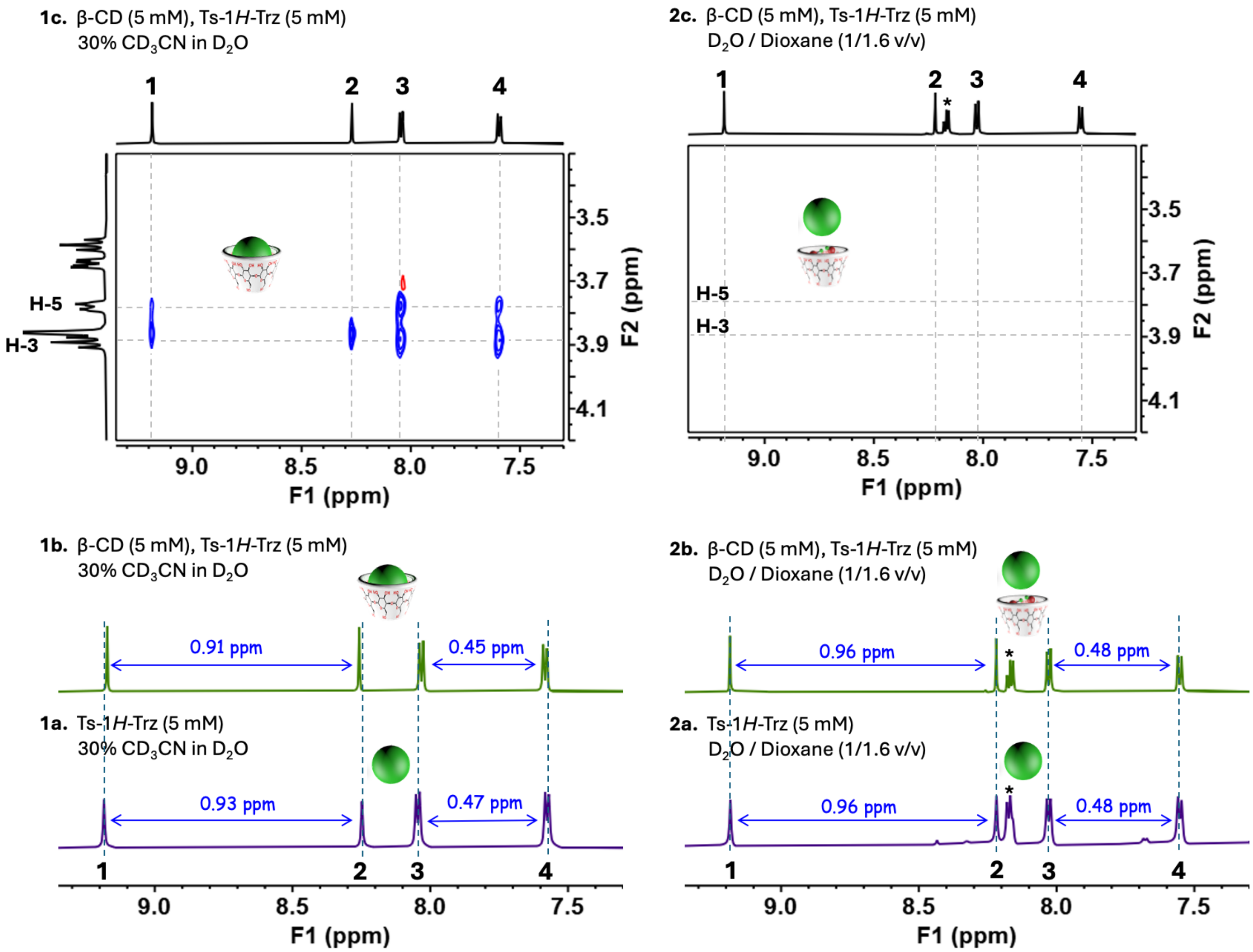

Fig. S11 ^{1}H NMR and 2D ROESY analysis of the interaction between β-CD (5mM) and Ts-1*H*-Trz (5mM) under two solvent systems: (**1**) in 30% CD_3CN in D_2O and (**2**) in D_2O/1,4-dioxane (1:1.6, v/v) (600 MHz, 25 °C). (**a**, **b**) Stacked ^{1}H NMR spectra of Ts-1*H*-Trz alone (**a**) and in the presence of β-CD (**b**); chemical-shift differences between the aromatic proton signals (peaks 1-4) are indicated. (**c**) Expanded 2D ROESY spectra (aromatic protons of Ts-1*H*-Trz (labeled 1-4) vs β-CD H-3 and H-5). In the CD_3CN/D_2O system (**1c**), cross-peaks are observed between the β-CD cavity protons (H-3, H-5) and the aromatic protons of Ts-1*H*-Trz, indicating host–guest complex formation. In the D_2O/1,4-dioxane system (**2c**), no such cross-peaks are observed, indicating the absence of complexation under the reaction conditions. 2D ROESY spectra were obtained at 25°C with mixing time of 250 ms. The asterisk (*) denotes a 1,4-dioxane impurity peak.

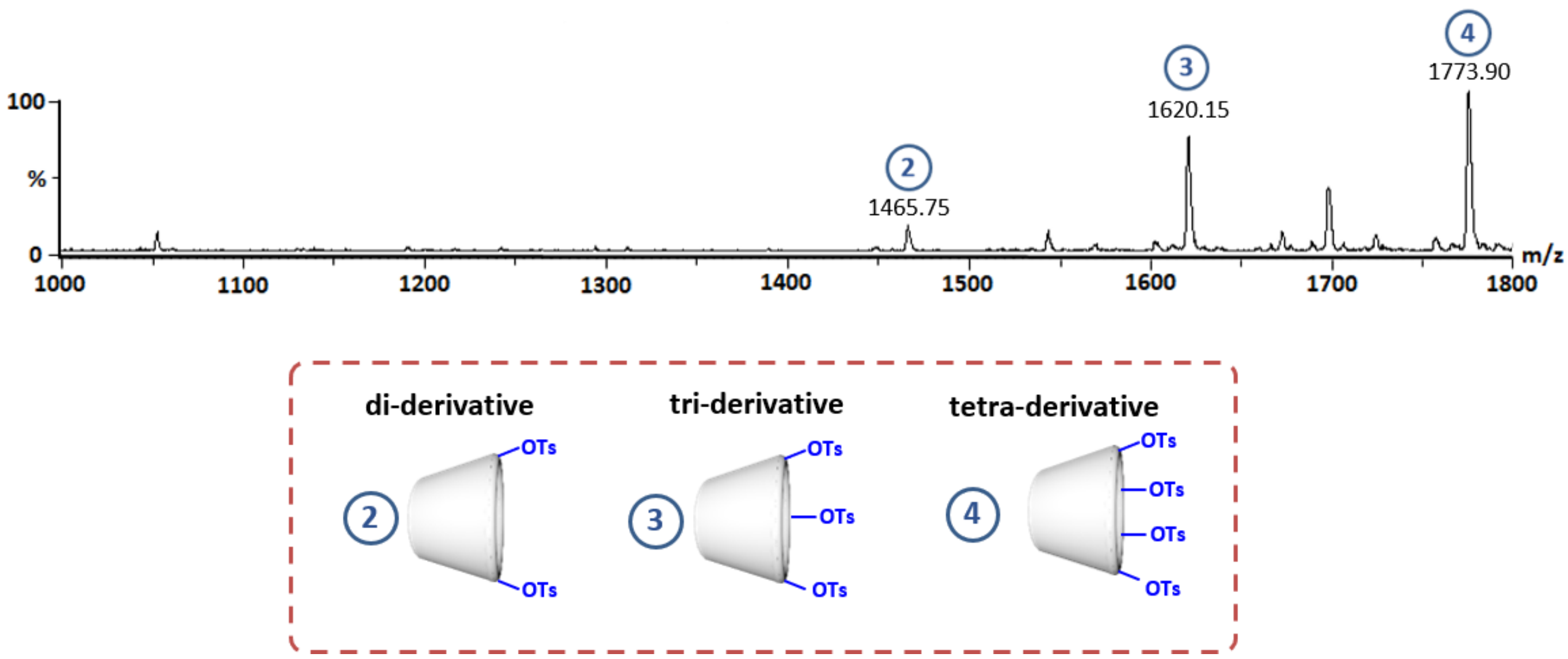

Fig. S12 ESI-MS spectrum of the trace insoluble material obtained during a representative synthesis of 2^{A}-*O*-Ts-β-CD, showing m/z values consistent with di-, tri- and tetra-tosylated β-CD species.

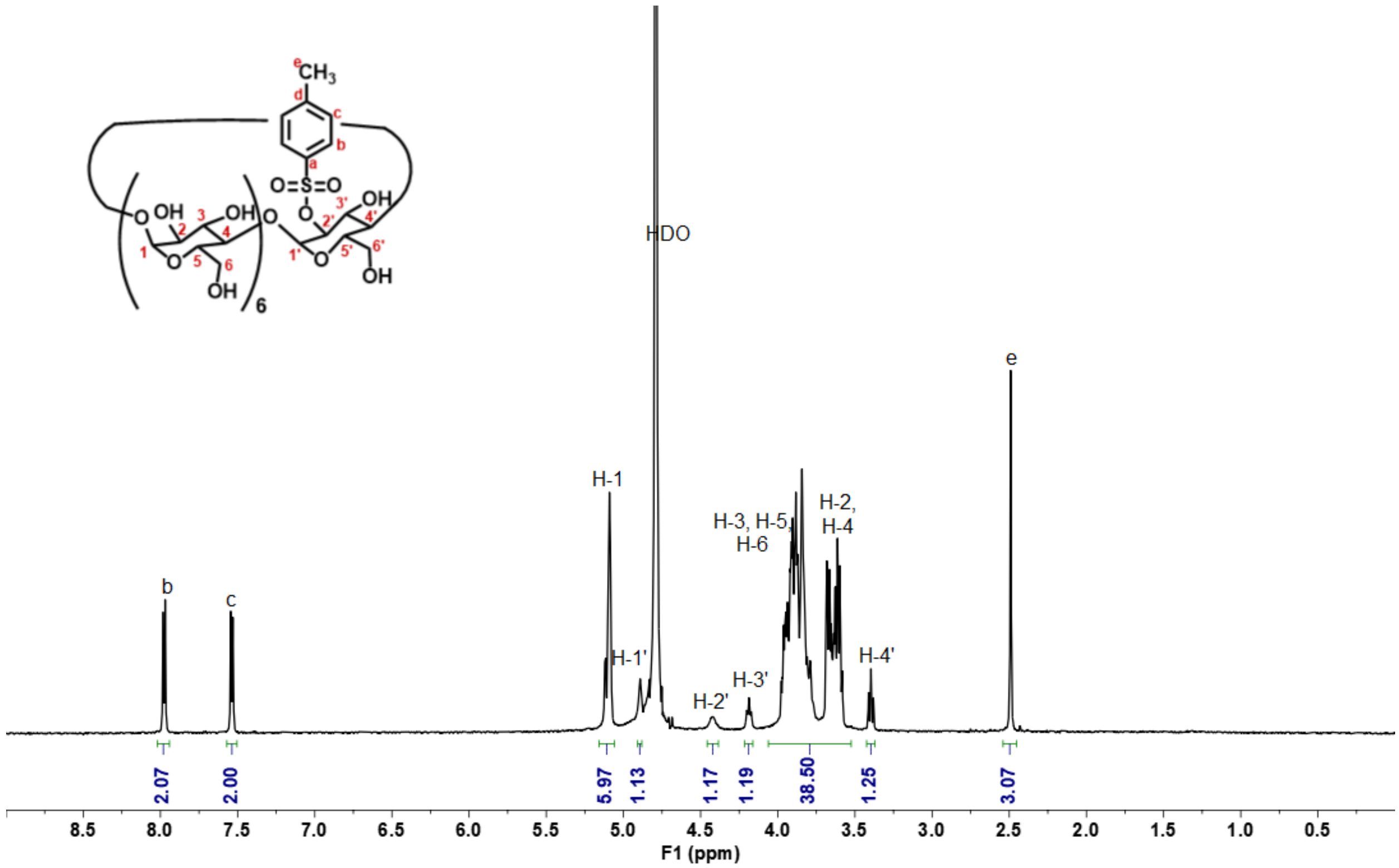

Fig. S13 ^{1}H NMR of 2^A-*O*-Ts-β-CD in D_2O (600 MHz, 25 °C).

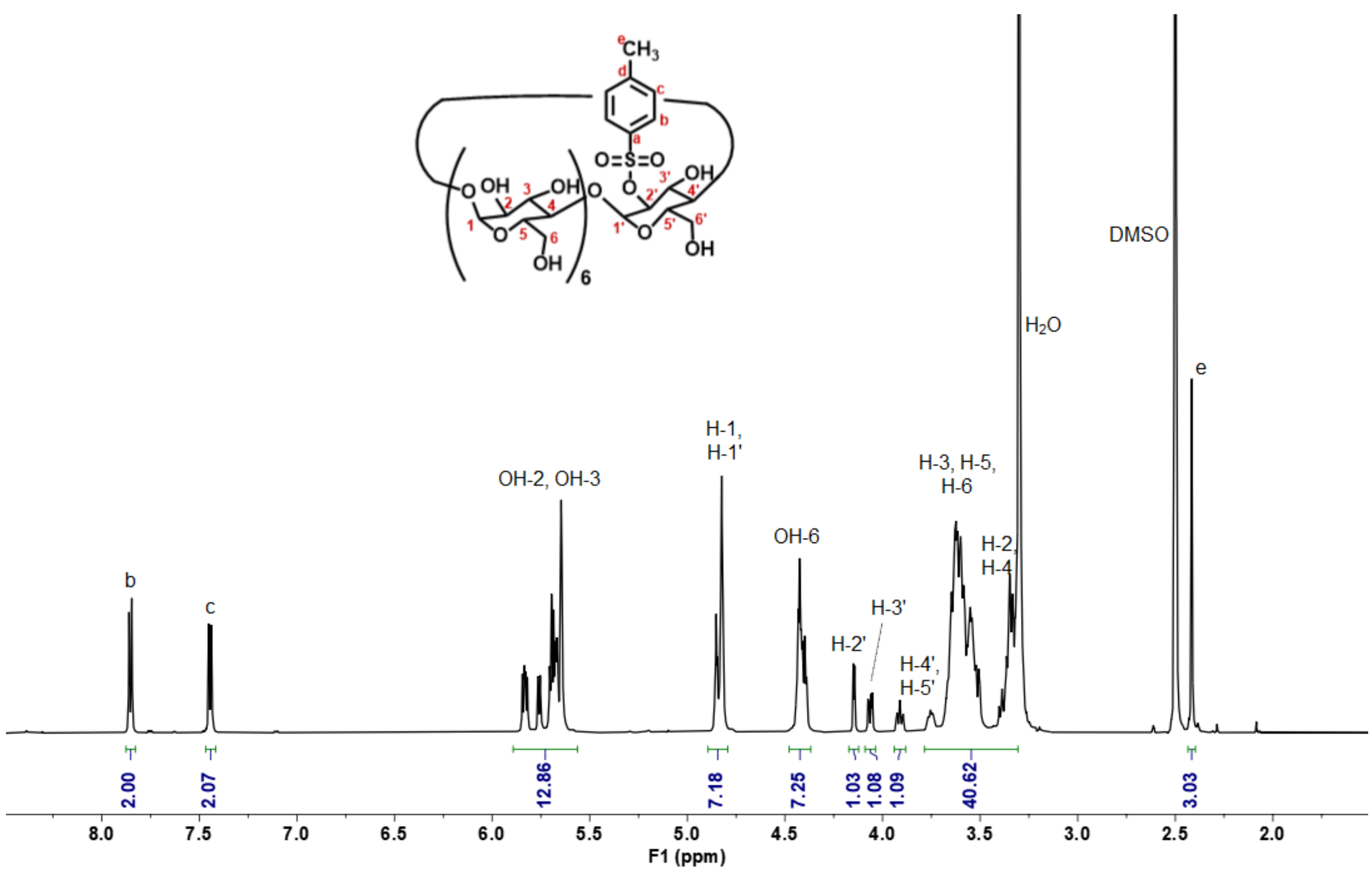

Fig. S14 ^{1}H NMR of 2^A-*O*-Ts-β-CD in DMSO-d_6 (600 MHz, 25 °C).

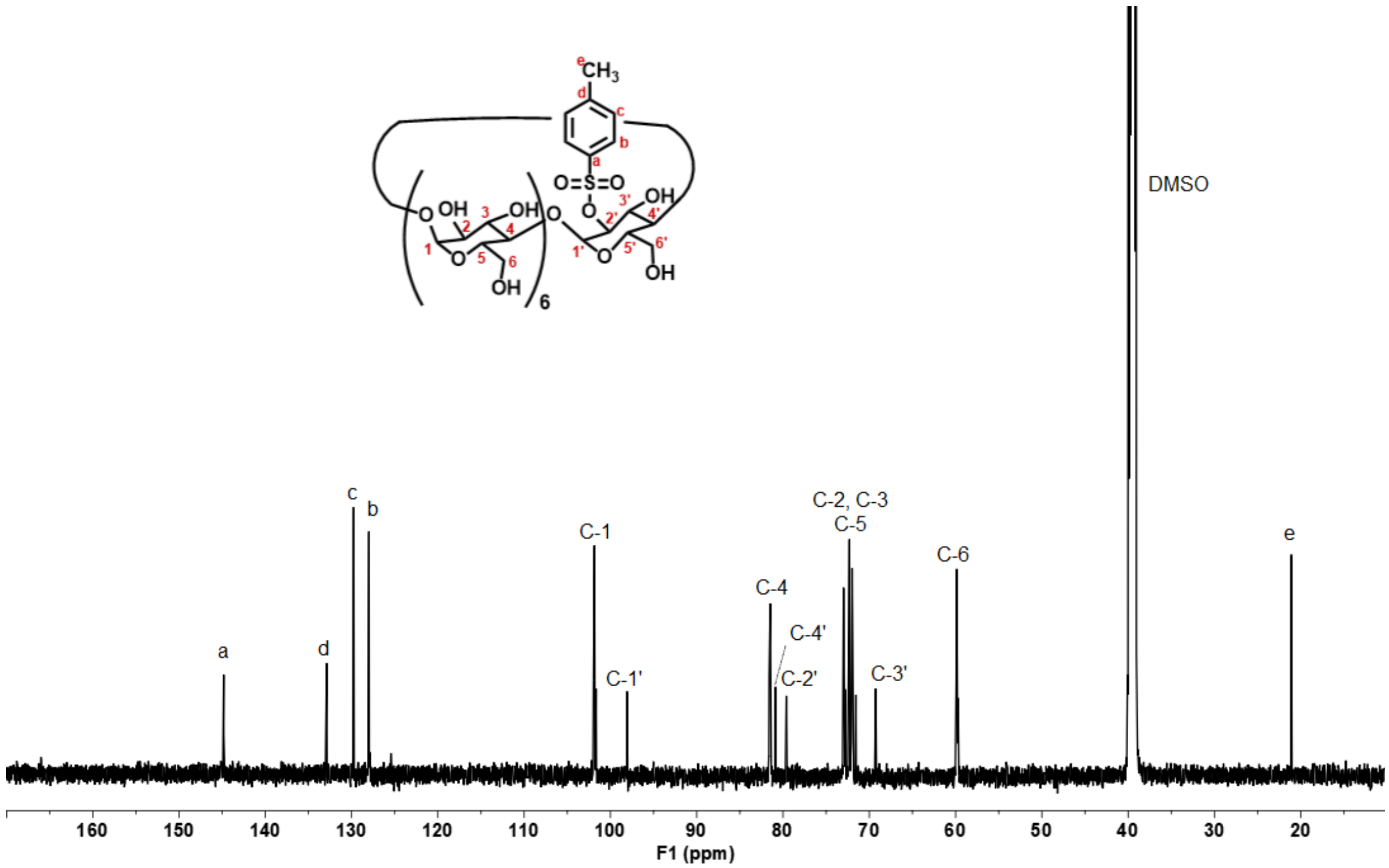

Fig. S15 ^{13}C NMR of 2[A]-*O*-Ts-β-CD in DMSO-d_6 (151 MHz, 25 °C).

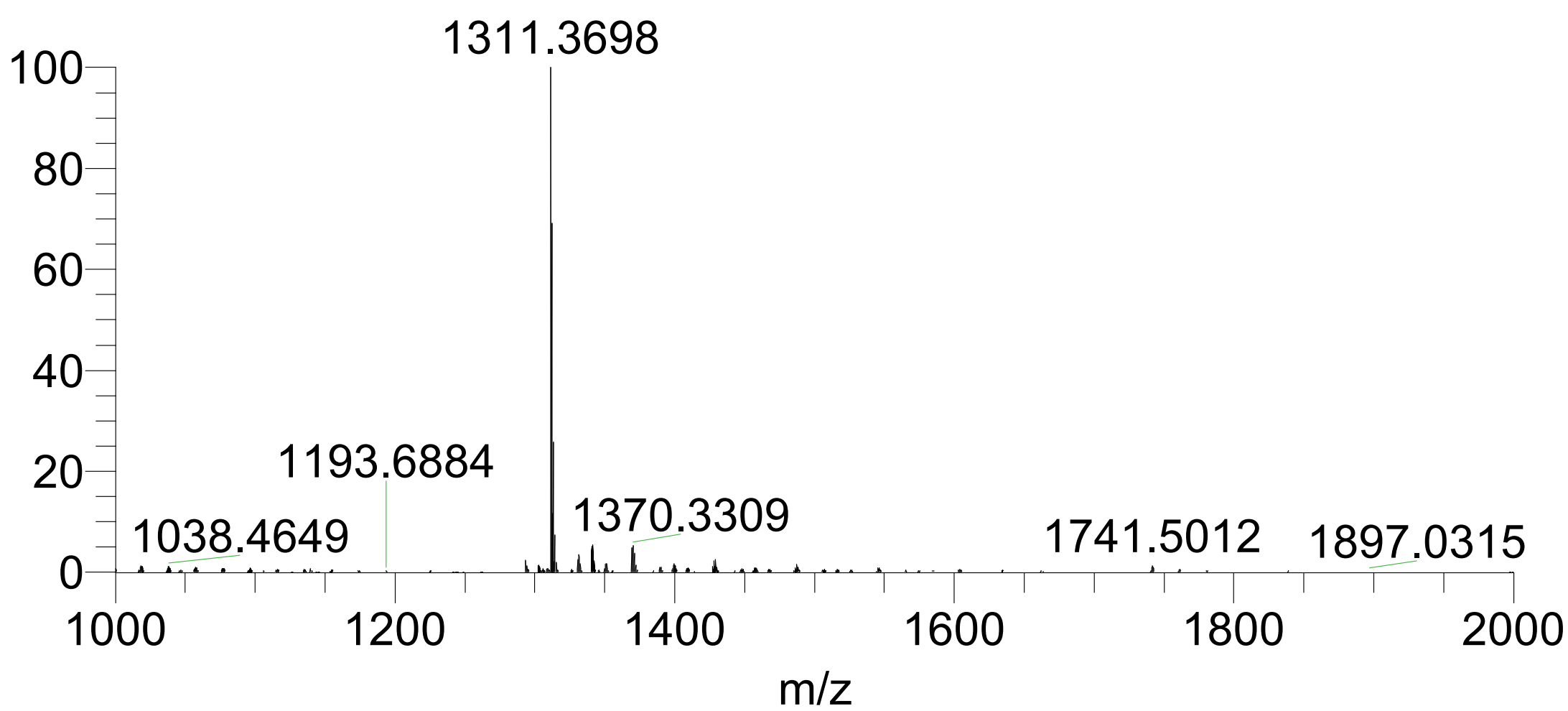

Fig. S16 HRMS (ESI) of 2[A]-*O*-Ts-β-CD m/z. Calculated for $C_{49}H_{76}NaO_{37}S$ $[M+Na]^+$ = 1311.3684, found 1311.3696.
